# Characterization of a temperate bacteriophage isolated from the ruminant-derived *Streptococcus bovis/equinus* complex

**DOI:** 10.1038/s41598-026-50797-4

**Published:** 2026-05-17

**Authors:** Seon Young Park, Jun-Seob Kim, Do Young Jin, Ye Bin Kim, Tae Seon Cha, Soojin Lim, Seunghui Lee, Seongwon Seo, Ji Hyung Kim

**Affiliations:** 1 Veterinary Drugs and Biologics Division, Animal and Plant Quarantine Agency, Gimcheon, 39660 Republic of Korea; 2 Division of Animal and Dairy Sciences, College of Agriculture and Life Science, Chungnam National University, Daejeon, 34134 Republic of Korea; 3 Department of Nano-Bioengineering, Incheon National University, Incheon, 22012 Republic of Korea; 4 Department of Food Science and Biotechnology, College of Bionano Technology, Gachon University, Seongnam, 13120 Republic of Korea; 5 Laboratory of Aquatic Biomedicine, College of Veterinary Medicine and Research Institute for Veterinary Science, Seoul National University, Seoul, 08826 Republic of Korea

**Keywords:** Temperate bacteriophage, *Streptococcus bovis/equinus* complex, Prophage, Ruminants, *Streptococcus* phage, Biotechnology, Microbiology

## Abstract

Although *Streptococcus bovis/equinus* complex (SBSEC) lysogens are prevalent in the rumen, studies on prophages experimentally induced from these strains are limited. Herein, we screened inducible prophages from ruminant-derived SBSEC strains, including the newly reported *S. ruminicola*, leading to the characterization of a representative temperate phage. We successfully induced a temperate phage (vB_SbS-proRumen) from *S. ruminicola* KCCM 90354, which has a broad host range, capable of lysing several SBSEC strains and other lactic acid bacteria. The vB_SbS-proRumen belongs to the Siphovirus, characterized by a short latent period, high burst size, and stability across pH and temperature conditions typical of subacute ruminal acidosis. The phage also demonstrates potent anti-biofilm activity. The vB_SbS-proRumen has a 38,092 bp double-stranded DNA genome with 58 predicted open reading frames, sharing high similarity and conserved genetic organization with other previously predicted SBSEC prophages. This study provides valuable insights into the diversity and plasticity of SBSEC phages.

**Supplementary Information:**

The online version contains supplementary material available at 10.1038/s41598-026-50797-4.

## Introduction

The *Streptococcus* (*S*.) *bovis*/*equinus* complex (SBSEC) comprises a group of lactic acid-producing bacteria commonly found in the gastrointestinal tract of humans and animals, as well as in dairy products, such as milk and cheese^1–3^. Our recent study proposed *S*. *ruminicola*, a new species isolated from ruminants, as an SBSEC member, exhibiting distinct biological and genomic properties distinguishing it from other strains within the complex^4^. Several species and subspecies within the SBSEC have been recognized as opportunistic pathogens that cause clinical infections, such as endocarditis and bacteremia in humans^5^; metabolic disorders, such as ruminal lactic acidosis in ruminants^6^; and mastitis in dairy cattle^7^. The extensive use of antimicrobials to treat and prevent bacterial infections has led to the emergence of antimicrobial-resistant (AMR) bacteria and the widespread dissemination of antibiotic-resistance genes (ARGs), which have become a global public health concern^8^. This issue is not limited to clinical isolates, particularly of known pathogens, as recent studies have shown that SBSEC strains, including *S. ruminicola*, isolated from non-clinical sources, such as ruminants and dairy products, also exhibit resistance to commonly used veterinary medicine, including tetracycline, erythromycin, and clindamycin, and harbor intrinsic resistance genes^9–11^. Although the prevalence of AMR in environmental SBSEC isolates is lower than that in clinical isolates, the presence of ARGs highlights the need to investigate alternative, safe approaches to control the spread of AMR bacteria and ARGs within SBSEC.

Bacteriophages (phages) are alternatives to traditional antibiotics and represent a promising approach to control AMR bacteria^12^. Phages are viruses that specifically infect bacteria and are primarily classified by two life cycles: lytic and lysogenic. Other lifestyles, including chronic infection and pseudolysogeny, have also been documented^13^. Lytic phages exhibit a lytic cycle in which they infect a specific host bacterial cell by injecting their genome. In contrast, temperate phages can alternate between the lytic and lysogenic cycles^14,15^. During the lysogenic cycle, the phage genome integrates into the bacterial chromosome to form a prophage; in certain cases, prophages may also persist as extrachromosomal elements^16^. The prophage remains dormant until induced by environmental factors, such as exposure to ultraviolet radiation or treatment with mitomycin C, triggering a transition to the lytic cycle^14,17^. The lysogenic cycle, characterized by iterative cycles of genome integration and excision, enables temperate phages to facilitate ARG transmission between different bacterial species and influences bacterial cellular properties, such as biofilm formation^18^. Hence, wild-type temperate phages are typically deemed unsuitable for direct inclusion in phage cocktails or use as biocontrol agents due to inherent risks of lysogeny. Nevertheless, advancements in genetic engineering have revealed new avenues for repurposing temperate phages by converting them into obligately lytic variants through the targeted deletion of lysogeny-related modules, such as integrase and repressor genes^19,20^. These engineered lytic derivatives have demonstrated an enhanced ability to limit the emergence of phage-resistant bacteria compared with naturally lytic phages alone^19^. To facilitate these strategies, a comprehensive characterization of temperate phages derived from lysogenic strains (lysogens) and evaluation of their potential benefits are essential.

Potential lysogens in SBSEC have been studied extensively, highlighting the high prevalence of temperate phages and lysogens isolated from ruminants^21–23^. To date, lytic and lysogenic phages infecting SBSEC have been identified as abundant viral groups in the rumen environment, with former morphologies indicative of Siphovirus and Podovirus^24,25^. In a previous study, we isolated two lytic SBSEC phages from ruminants, vB_SbRt-pBovineB21 and vB_SbRt-pBovineS21. These phages exhibit significant lytic activity and anti-biofilm properties against SBSEC species and various lactic acid-producing bacteria, indicating their potential as novel biocontrol agents^25^. Recent advances in sequence-based approaches have revealed that certain putative prophage regions are integrated into the genome of SBSEC isolates, demonstrating the prevalence of prophages within SBSEC strains^26,27^. Despite these findings, the characteristics of temperate phages in SBSEC strains, particularly those harboring prophage regions that can be induced to enter the lytic cycle, remain largely unexplored.

Therefore, the primary objectives of this study were to (i) screen the inducible prophage-possessing SBSEC strains, (ii) identify a susceptible SBSEC strain (indicator host) for infecting and amplifying an induced prophage, (iii) compare genetic characteristics between the integrated prophage in the SBSEC genome and the amplified temperate phage, and (iv) comprehensively characterize the biological and genomic properties of a representative SBSEC temperate phage, designated vB_SbS-proRumen, employing an in vitro experimental system.

## Results

### SBSEC prophage induction and indicator host determination of SBSEC Phage vB_SbS-proRumen

**Fig. 1:**
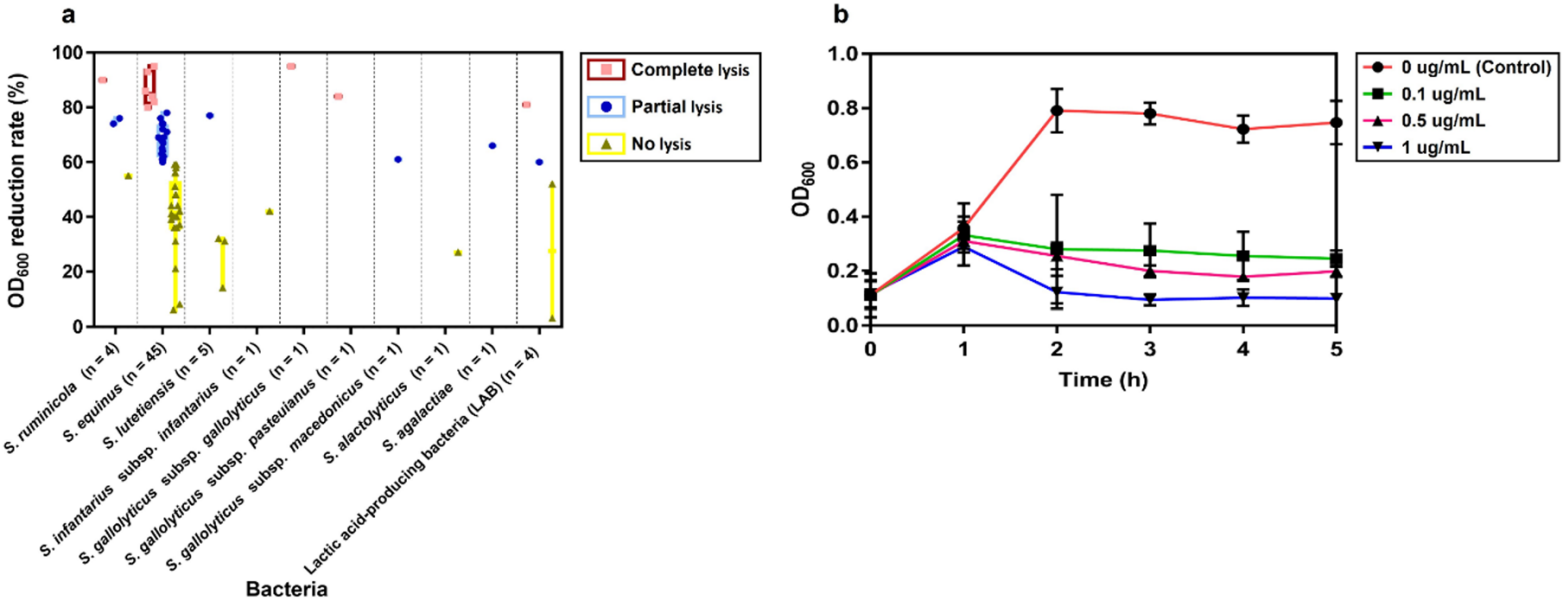
Induction features in SBSEC isolates and gram-positive-type strains. (**a**) Prophage induction screening using 1 µg/mL mitomycin C (MMC). All strains were treated with MMC at the early exponential phase, and categorized into three lysis patterns based on OD_600_ reduction after MMC treatment compared to their respective untreated control: complete lysis (> 80% reduction), partial lysis (60–80% reduction), and no lysis (< 60% reduction). (**b**) Growth curves of the SBSEC lysogenic strain KCCM 90354 treated with different concentrations of MMC after incubation for 5 h. MMC was added to early exponential phase cultures at three concentrations: 0.1, 0.5, and 1 µg/mL. The experiments were performed in triplicate; error bars represent the standard deviation.

Inducible prophages were screened in 65 bacterial strains using mitomycin C (MMC; Sigma-Aldrich, St. Louis, MO, USA), a well-known prophage-inducing agent (Fig. 1a and **Fig. S1–S3**). For initial screening, the strains were classified into three distinct lysis patterns based on differences in final OD_600_ values between MMC-treated and untreated cultures. Complete lysis (> 80% reduction in OD_600_) was observed in ten strains (15.3%), including *S*. *ruminicola* isolates, six *S. equinus* isolates, and type strains of *S*. *gallolyticus* subsp. *gallolyticus*, *S*. *gallolyticus* subsp. *pastuerianus*, and *Lactilactobacillus* (*L*.) *sakei*. Partial lysis (60–80% reduction in OD_600_) was observed in 24 strains (36.9%), including 18 *S. equinus* strains, two *S*. *ruminicola* strains, one *S*. *lutetiensis*, and one type from the *S*. *gallolyticus* subsp. *macedonicus*, *S*. *agalactiae*, and *Lacticaseibacillus* (*Lc*.) *casei* strains. The remaining strains (50%) showed no lysis (< 60% reduction in OD_600_) compared with the untreated controls.

To confirm the presence of induced prophages in *S*. *ruminicola* KCCM 90354, a plaque assay was performed using the cell-free supernatant obtained from MMC-treated cultures. The bacterial inoculums were subjected to prophage induction using three MMC concentrations (0.1, 0.5, and 1 µg/mL). A significant reduction in OD_600_ was observed in all treated cultures compared with the untreated control, suggesting successful prophage induction (Fig. 1b).

The presence of infectious phage particles in the supernatant was confirmed by plaque formation on the lawn of indicator strains using the double-agar method. The host range of the induced SBSEC temperate phage, designated vB_SbS-proRumen, was determined by assessing its ability to form plaques on 65 bacterial strains, with efficiency of plating (EOP) calculated for each susceptible strain. Of these, 16 strains—including *S*. *ruminicola*, *S. equinus*, *S*. *lutetiensis*, *Lc*. *casei* ATCC 393^T^, and *L. sakei* ATCC 15521^T^—were susceptible (Table 1). Ten strains exhibited clear plaques with relatively high EOP values (0.64–0.81), six had turbid plaques with lower EOP values (0.26–0.57), likely due to partial lysis or inhibition from lysogenic conversion-related factors in the supernatant. None of the nine SBSEC type strains were susceptible. EOP data confirmed productive phage infection in all plaque-forming strains, indicating lytic zones resulted from phage replication rather than residual mitomycin C or free endolysin activity.

**Table 1:** Host spectrum of the SBSEC temperate phage vB_SbS-proRumen used in this study.

Bacterial species	Isolate source	Number of bacteria infected by phage vB_SbS-proRumen	Strain	Plaque morphology	EOP	References
*S. ruminicola*	*Bos taurus coreanae*	1/2	KCCM 90356	Clear	0.80	^9^
	*Capra aegagrus hircus*	0/1	–	Absent	–	
*S. equinus*	*Bos taurus*	1/13	KCCM 90359	Clear	0.69	^9^
	*Bos taurus coreanae*	4/17	KCCM 90377	Clear	0.70	
			KCCM 90383	Clear	0.74	
			KCCM 90384	Clear	1	
			KCCM 90385	Turbid	0.38	
	*Capra aegagrus hircus*	6/14	KCCM 90386	Clear	0.65	
			KCCM 90388	Clear	0.81	
			KCCM 90389	Clear	0.64	
			KCCM 90390	Turbid	0.51	
			KCCM 90399	Turbid	0.57	
			KCCM 90400	Clear	0.70	
*S. lutetiensis*	*Capra aegagrus hircus*	2/4	KCCM 90393	Clear	0.67	^9^
			KCCM 90397	Clear	0.69	
Type strains of SBSEC						
*S. ruminicola*	*Bos taurus*	0/1	KCTC 43308^T^	Absent	–	KCTC
*S. equinus*	*Equus ferus caballus*	0/1	ATCC 9812^T^	Absent	–	ATCC
*S. bovis*	*Bos taurus*	0/1	ATCC 33317^T^	Absent	–	ATCC
*S. infantarius* subsp. *Infantarius*	*Homo sapiens*	0/1	ATCC BAA-102^T^	Absent	–	ATCC
*S. gallolyticus* subsp. *Gallolyticus*	*Phascolarctos cinereus*	0/1	CCUG 35224^T^	Absent	–	CCUG
*S. gallolyticus* subsp. *Pastuerianus*	*Homo sapiens*	0/1	CCUG 46150^T^	Absent	–	CCUG
*S. gallolyticus* subsp. *Macedonicus*	Cheese	0/1	ATCC BAA-249^T^	Absent	–	ATCC
*S. lutetiensis*	*Homo sapiens*	0/1	CCUG 46149^T^	Absent	–	CCUG
*S. alactolyticus*	*Sus domesticus*	0/1	ATCC 43077^T^	Absent	–	ATCC
*S. agalactiae*	*Homo sapiens*	0/1	ATCC 13813^T^	Absent	–	ATCC
*Lacticaseibacillus casei*	Cheese	1/1	ATCC 393^T^	Turbid	0.38	ATCC
*Lactiplantibacillus plantarum* subsp. *plantarum*	Cabbage	0/1	ATCC 14917^T^	Absent	–	ATCC
*Latilactobacillus sakei*	Yeast starter	1/1	ATCC 15521^T^	Turbid	0.26	ATCC
*Lactococcus lactis* subsp. *lactis*	Unknown	0/1	ATCC 19435^T^	Absent	–	ATCC

*T: Type strain.

### Biological characteristics of SBSEC phage vB_SbS-proRumen

#### Morphology and bacteriolytic activity

**Fig. 2:**
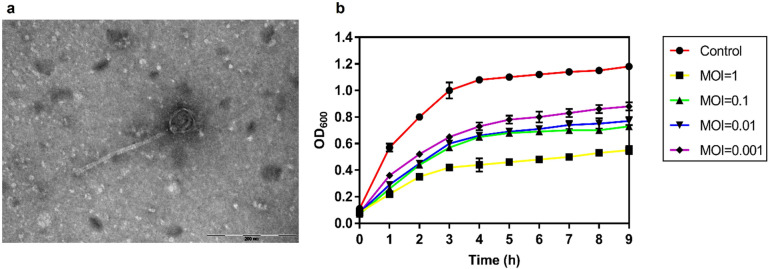
Morphological and functional characterization of the SBSEC temperate phage vB_SbS-proRumen. (**a**) Transmission electron micrograph of the SBSEC temperate phage vB_SbS-proRumen. Scale bar = 200 nm. (**b**) Bacteriolytic activity of vB_SbS-proRumen against indicator host *S*. *ruminicola* KCCM 90384. The experiments were performed in triplicate; error bars represent standard deviation.

Transmission electron microscopy (TEM) analysis of vB_SbS-proRumen revealed a morphology consistent with that of Siphovirus, exhibiting an icosahedral head with a 47.3 ± 2.1 nm diameter and a 222.8 ± 1.8 nm long non-contractile tail (Fig. 2a). The phage titer on the indicator host strain, *S*. *ruminicola* KCCM 90384, reached a maximum of approximately 10^8^ plaque-forming units (PFU)/mL. To evaluate the bacteriolytic activity of vB_SbS-proRumen against its indicator host strain, bacterial growth inhibition was monitored at various multiplicities of infection (MOIs; 0.001, 0.01, 0.1, and 1; Fig. 2b). The phage exhibited moderate inhibition of bacterial growth across all tested MOIs; however, complete lysis of the bacterial culture was not observed under any condition. This incomplete lysis aligned with the temperate nature of the phage, as a proportion of host cells likely underwent lysogenization rather than lytic infection. The degree of inhibition was MOI-dependent, with a notable reduction in bacterial growth observed at an MOI of 1, 2 h post-inoculation, compared with lower MOIs and the control without phage treatment.

#### Adsorption rate, one-step growth curve, and stability

**Fig. 3:**
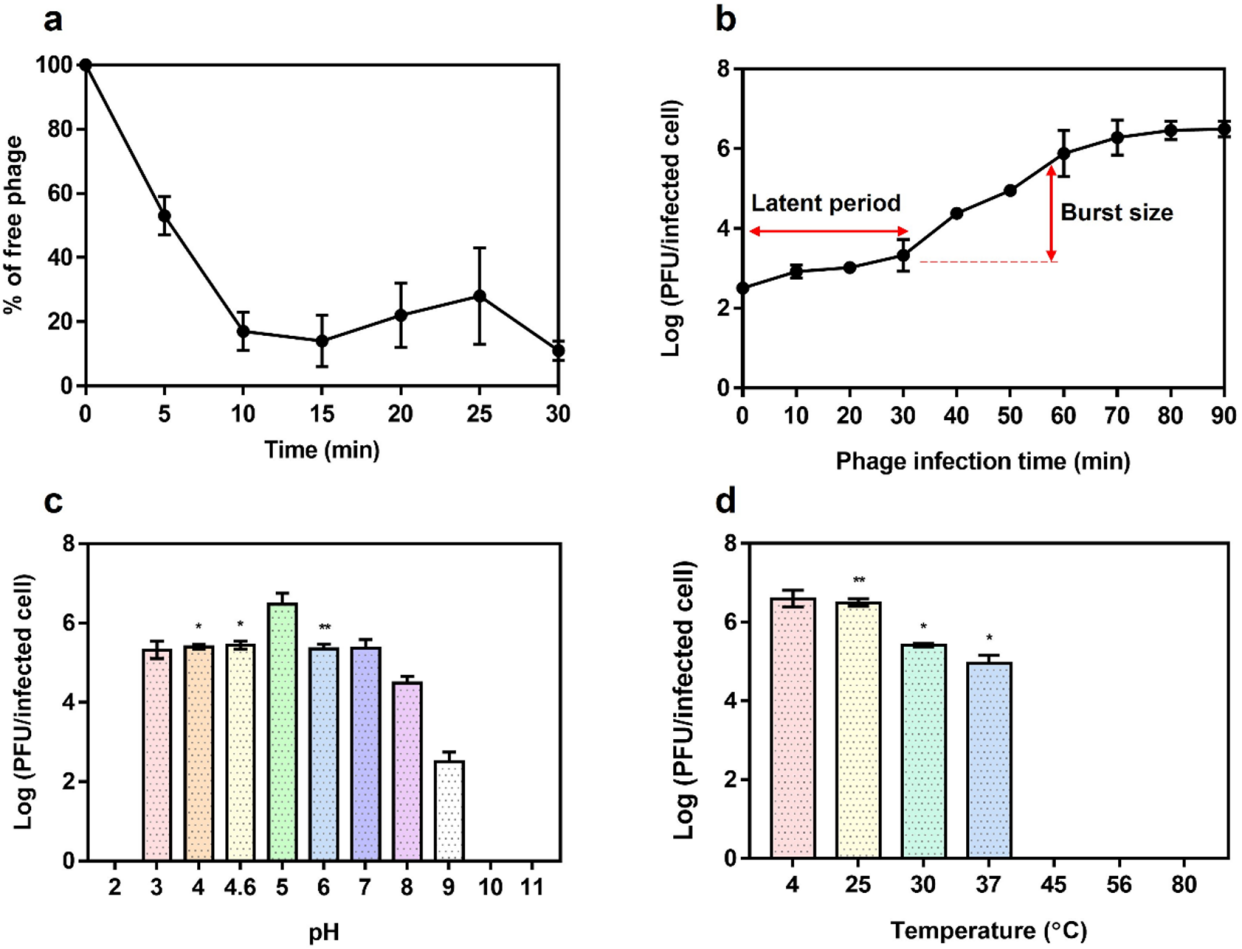
Biological characteristics of the SBSEC temperate phage vB_SbS-proRumen. Adsorption kinetic (**a**) and one-step growth (**b**) curves of the phage on indicator host *S*. *ruminicola* KCCM 90384. pH (**c**) and thermal (**d**) stability of the phage under different conditions. The phage was incubated at indicated pH values for 3 h at 37 °C, or at the indicated temperatures for 3 h, respectively. The pH 7 condition (**c**) and 4 °C condition (**d**) represent the initial phage titers used as the respective controls. The detection limit of the plaque assay was 10^1^ PFU/mL. The experiments were performed in triplicate; error bars represent standard deviation. *p < 0.05 and **p < 0.01 compared with the respective controls (pH 7 for pH stability; 4 °C for thermal stability), as determined by one-way ANOVA followed by Dunnett’s multiple-comparison test.

To understand the life cycle of vB_SbS-proRumen and evaluate its efficiency against the indicator host strain KCCM 90384, adsorption and one-step growth assays were conducted. The adsorption assay, performed at an MOI of 0.01, revealed that approximately 80% of the phage particles were adsorbed by strain KCCM 90384 within 10 min at 37 °C (Fig. 3a). Based on the optimal adsorption time of 10 min, a one-step growth curve analysis was performed to determine the latent period and burst size of vB_SbS-proRumen. The results showed a latent period of 30 min, followed by a 30 min burst period, during which an average of 636.6 ± 5.2 PFUs were released per infected cell (Fig. 3b).

To assess the adaptability of vB_SbS-proRumen to various environmental conditions, stability assays were conducted under different pH and thermal conditions. The phage was stable over a wide pH range, from 3 to 8, with only slightly decreased activity at pH 9. However, the phage was completely inactivated under extremely acidic (pH 2) and basic (pH > 10) conditions (Fig. 3c). Temperature stability assays revealed that the phage was relatively stable at 4, 25, 30, and 37 °C; however, it was completely inactivated at temperatures above 45 °C (Fig. 3d).

#### Anti-biofilm activity

**Fig. 4:**
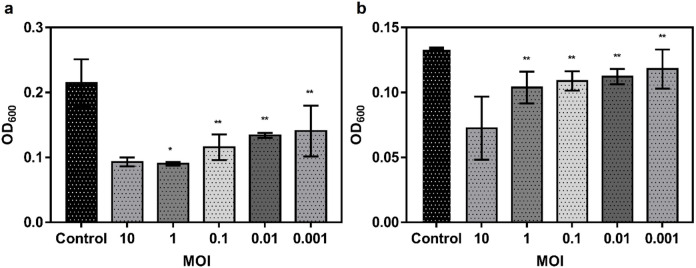
Anti-biofilm activity of the SBSEC temperate phage vB_SbS-proRumen at MOIs of 0.001, 0.01, 0.1, 1, and 10. (**a**) Prevention of biofilm formation: *S*. *ruminicola* KCCM 90384 was co-cultured with vB_SbS-proRumen at various MOIs for 24 h at 37 °C. (**b**) Degradation of pre-formed biofilms: mature biofilms of strain KCCM 90384 were formed for 24 h at 37 °C, followed by treatment with vB_SbS-proRumen at different MOIs for an additional 24 h. In both assays, the biofilm biomass was quantified using crystal violet staining and measured by determining the OD_600_. The experiments were performed in triplicate; error bars represent the standard deviation. *p < 0.05 and **p < 0.01 compared to the respective controls, as determined by one-way ANOVA followed by Dunnett’s multiple-comparison test.

The anti-biofilm activity of vB_SbS-proRumen was evaluated by preventing biofilm formation and degrading pre-formed biofilms. The experiments were conducted at MOIs of 0.001, 0.01, 0.1, 1, and 10. The biofilm mass was quantified by measuring the OD_600_ using the crystal violet (CV) staining method. In the biofilm prevention assay, the phage significantly inhibited biofilm formation by *S*. *ruminicola* KCCM 90384 in an MOI-dependent manner (Fig. 4a). The OD_600_ values of all phage-treated groups decreased to < 0.14 compared with the untreated control, with higher MOIs resulting in greater biofilm formation inhibition. Similarly, in the biofilm degradation assay, the phage effectively reduced the biomass of pre-formed mature biofilms in an MOI-dependent manner (Fig. 4b). The OD_600_ values of all phage-treated groups decreased to < 0.12 compared with the untreated control, with higher MOIs resulting in greater biofilm degradation.

### Genomic characteristics of prophage-inducible SBSEC strain *S*. *ruminicola* KCCM 90354 and temperate phage vB_SbS-proRumen

#### Genomic analysis of *S*. *ruminicola* KCCM 90354

**Fig. 5:**
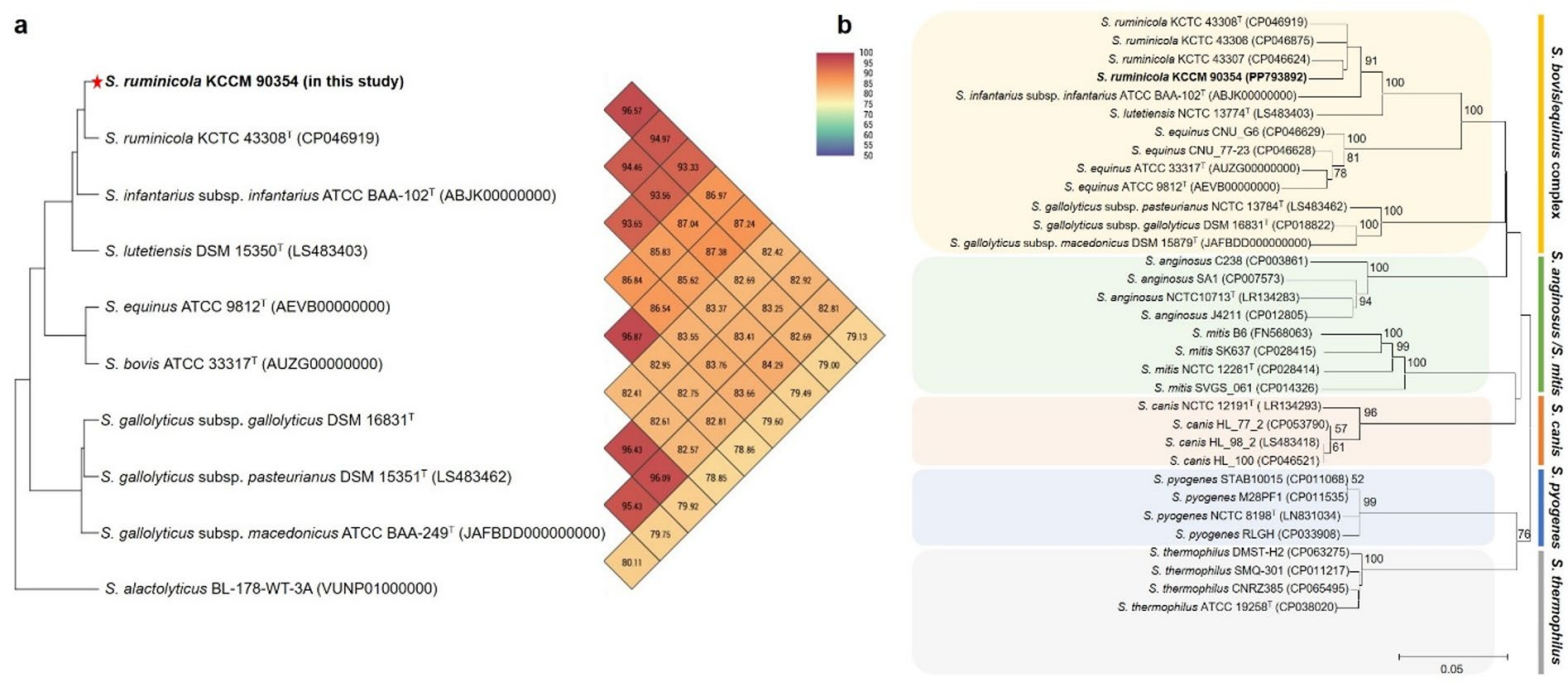
Genomic relationships of *S*. *ruminicola* KCCM 90354 with SBSEC type strains. (**a**) Heatmap of Orthologous Average Nucleotide Identity (orthoANI) values for the *S*. *ruminicola* KCCM 90354 genome compared with those of nine SBSEC type strains. The values show similarity (%) among the genomes, calculated using OAT software. (**b**) Phylogenetic tree constructed with *Streptococcus* species, including eight SBSEC type strains, using the Type (Strain) Genome Server (TYGS). Two phylogenies with branch lengths scaled to GBDP distances (D6, VICTOR; D5, TYGS) are shown, and the numbers above branches are GBDP pseudo-bootstrap support values > 50% from 1,000 replications.

The SBSEC lysogenic strain KCCM 90354, which demonstrated high prophage induction ability, was previously identified based on the *sodA* gene^9^. Genomic analysis was conducted to determine whether the lysogen genome harbored a prophage. The lysogen genome comprised a circular DNA of 1,979,605 bp with a GC content of 37.7%, comprising 1,920 protein-coding genes, 71 tRNA genes, and 21 rRNA genes (**Fig. S4**). Moreover, the genomic analysis revealed the absence of ARGs and mobile genetic elements (MGEs), whereas one Type II-A CRISPR-Cas system was identified. Notably, none of the 25 spacers matched the prophage region. In addition, several virulence factors (VFs) related to adherence, enzyme, immune evasion, protease, bile resistance, and lipid and fatty acid metabolism were identified (**Table S1**). In the lysogen genome, a putative prophage region (1,433,081–1,471,088 bp) and its putative functional protein-coding genes were predicted using PHASTER (**Table S2**). To establish the genome-based identification of strain KCCM 90354 within SBSEC, Orthologous Average Nucleotide Identity (orthoANI) values were estimated and compared with those of nine SBSEC type strains. The strains clustered with *S*. *ruminicola* KCTC 43308^T^ (96.6%), *S*. *infantarius* subsp. *infantarius* ATCC BAA-102^T^ (95%), and *S*. *lutetiensis* DSM 15350^T^ (93.3%), indicating a close relationship with *S*. *ruminicola*, a recently recognized member of the SBSEC^4^ (Fig. 5a). This classification was corroborated by genome-based phylogenetic analysis using Type (Strain) Genome Server (TYGS), which consistently clustered strain KCCM 90354 with other SBSEC type strains (Fig. 5b). These findings suggest that *S*. *ruminicola* KCCM 90354 is a lysogen carrying a prophage within its genome. A prophage region was detected at positions 1,433,323–1,471,397 bp on the lysogen chromosome, flanked by attachment sites attL (5’-TGGATATTTACG-3’; positions 1,433,081–1,433,092) and attR (5’-TGGATATTTACG-3’; positions 1,471,077–1,471,088). The presence of identical attL and attR sequences confirmed site-specific integration mediated by the phage-encoded integrase.

#### Genomic features of temperate phage vB_SbS-proRumen

**Fig. 6:**
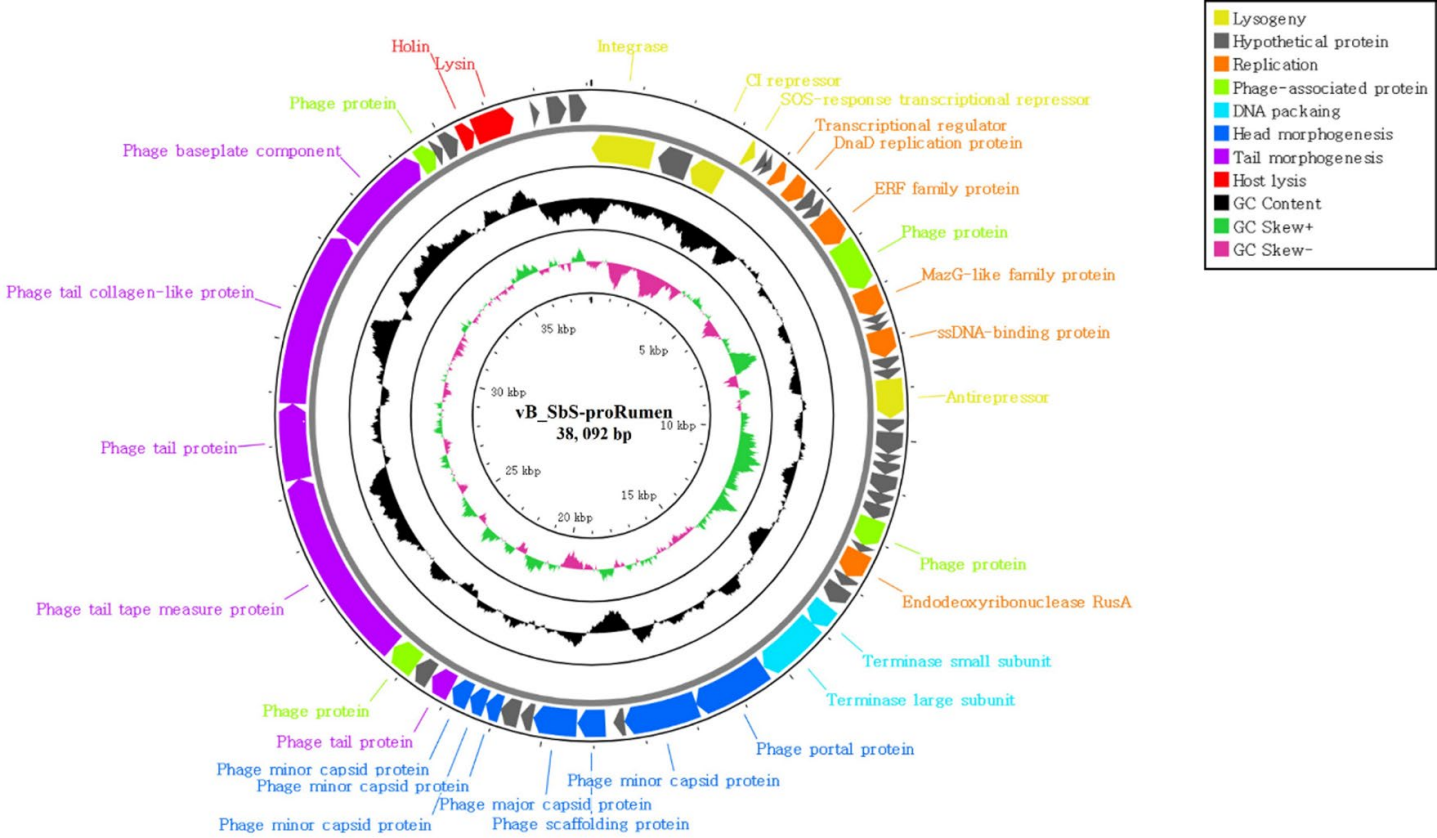
Circular genome map of SBSEC temperate phage vB_SbS-proRumen. Genomic map of the phage generated using Proksee, showing representations of CDSs in different color categories: putative predicted ORFs; lysogen (yellow), hypothetical protein (gray), replication (orange), phage-associated protein (light-green), DNA packaging (sky-blue), head morphogenesis (blue), tail morphogenesis (purple), host lysis (red); GC content (black); GC skew+(green); GC skew-(magenta).

The double-stranded DNA genome of the SBSEC temperate phage vB_SbS-proRumen was 38,092 bp with a 38.8% GC content and 58 putative ORFs (55 on the positive strand and three on the negative strand; Fig. 6). The predicted amino acid sizes of the annotated ORFs ranged from 47 to 1,294, with corresponding putative Mws of 5.3 to 132.4 kDa and pI between 4.1 and 10.2. Among the 58 ORFs, 56 had ATG start codons, whereas two (ORFs 7 and 56) had TTG start codons. No tRNA genes, VFs, or ARGs were detected in the phage genome. Functional prediction using BLASTp allowed the categorization of the 58 ORFs into eight functional groups: lysogeny (ORFs 1, 3, 4, 7, 19, and 57), replication (ORFs 8, 11, 13, 16, 17, 18, 29, and 58), phage-associated proteins (ORFs 12, 27, 46, and 51), DNA packaging (ORFs 32, 33, and 34), head morphogenesis (ORFs 35, 37, 38, 41, 42, and 43), tail morphogenesis (ORFs 44, 47, 48, 49, and 50), host lysis (ORFs 54 and 55), and hypothetical proteins (**Table S3**). Excluding ORFs 27 and 56, all predicted ORFs showed high homology (70.5–100% identity) with prophage-associated proteins identified in the SBSEC strains. BLASTn analysis revealed that the genome of vB_SbS-proRumen had the highest similarity to the four previously reported SBSEC prophage regions: *Streptococcus* phage Javan 210 (MK448875; query coverage, 67%; identity, 97.4%), *Streptococcus* phage Javan 206 (MK448874; query coverage, 74%; identity, 93.3%), *Streptococcus* phage Javan 284 (MK448898; query coverage, 60%; identity, 90%), and *Streptococcus* phage Javan 224 (MK448878; query coverage, 37%; identity, 82.1%). The prevalence of vB_SbS-proRumen-like prophages within SBSEC was evaluated through BLASTn analysis against 806 publicly available SBSEC genomes. Prophage regions exhibiting greater than 70% nucleotide identity to vB_SbS-proRumen were detected in 27 out of 806 strains (3.4%), reflecting a limited yet notable distribution of this prophage type among SBSEC. Subsequent PHASTER-based analysis of these 27 strains enabled prediction of the attachment sites associated with homologous prophage regions (**Table S4**), providing valuable insights into the conservation of integration mechanisms across related SBSEC prophages.

#### Phylogeny and comparative analysis of phage genome

##### Genome-based phylogenetic analyses

**Fig. 7:**
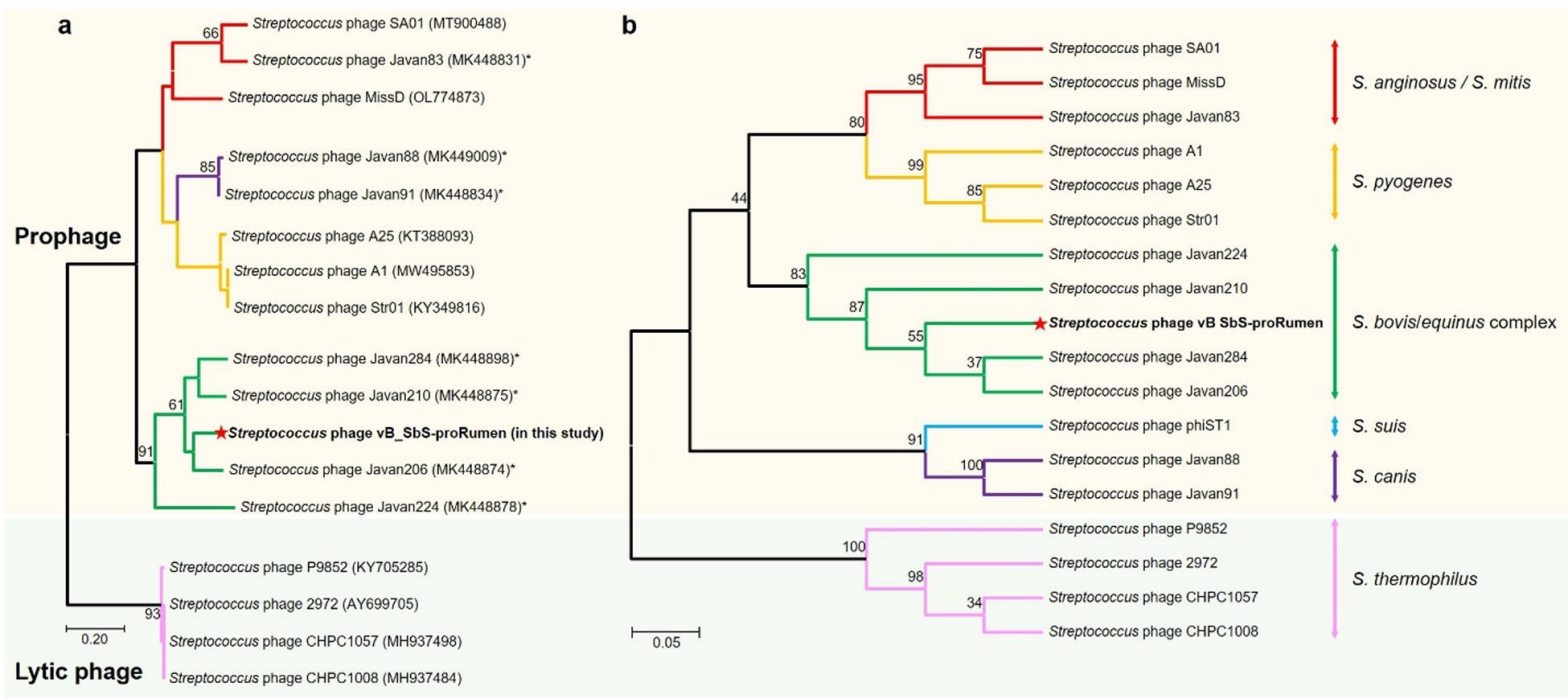
Phylogenetic relationships of the temperate phage vB_SbS-proRumen. (**a**) Whole-genome-based phylogenetic tree constructed using 17 *Streptococcus* phages (13 putative prophage regions and four lytic phages) retrieved from the GenBank database, generated using the Virus Classification and Tree Building Online Resource (VICTOR). The tree distinguishes prophage-derived sequences and lytic phages, as indicated. (**b**) Phylogenetic tree based on amino acid sequences of the terminase large subunit (TerL), inferred using the maximum-likelihood method with 1,000 bootstrap replicates in MEGA X. Clades are highlighted with matching colors according to consistent clustering patterns observed in both trees and correspond to host-associated *Streptococcus* species groups. Scale bars indicate the number of substitutions per site. *Streptococcus* prophage sequences correspond to predicted genomic regions deposited in the GenBank database and do not represent experimentally-induced phage isolates.

Several phylogenetic analyses of SBSEC temperate phage vB_SbS-proRumen were conducted with closely related *Streptococcus* phages using BLASTn (**Table S5**). The genome-based phylogeny (Fig. 7a) revealed that vB_SbS-proRumen clustered with previously predicted SBSEC prophages, yet was distinctly separated from previously isolated lytic *Streptococcus* phages. The phylogenetic tree illustrates each phage’s lytic or prophage classification and host species. Similarly, the terminase large subunit (TerL)-based phylogeny (Fig. 7b) aligned broadly with the genome-based findings; however, vB_SbS-proRumen formed a distinct branch from prophage Javan 206, which exhibited the highest similarity in the genome analysis.

##### Identification of the prophage region and gene-level comparison

**Fig. 8:**
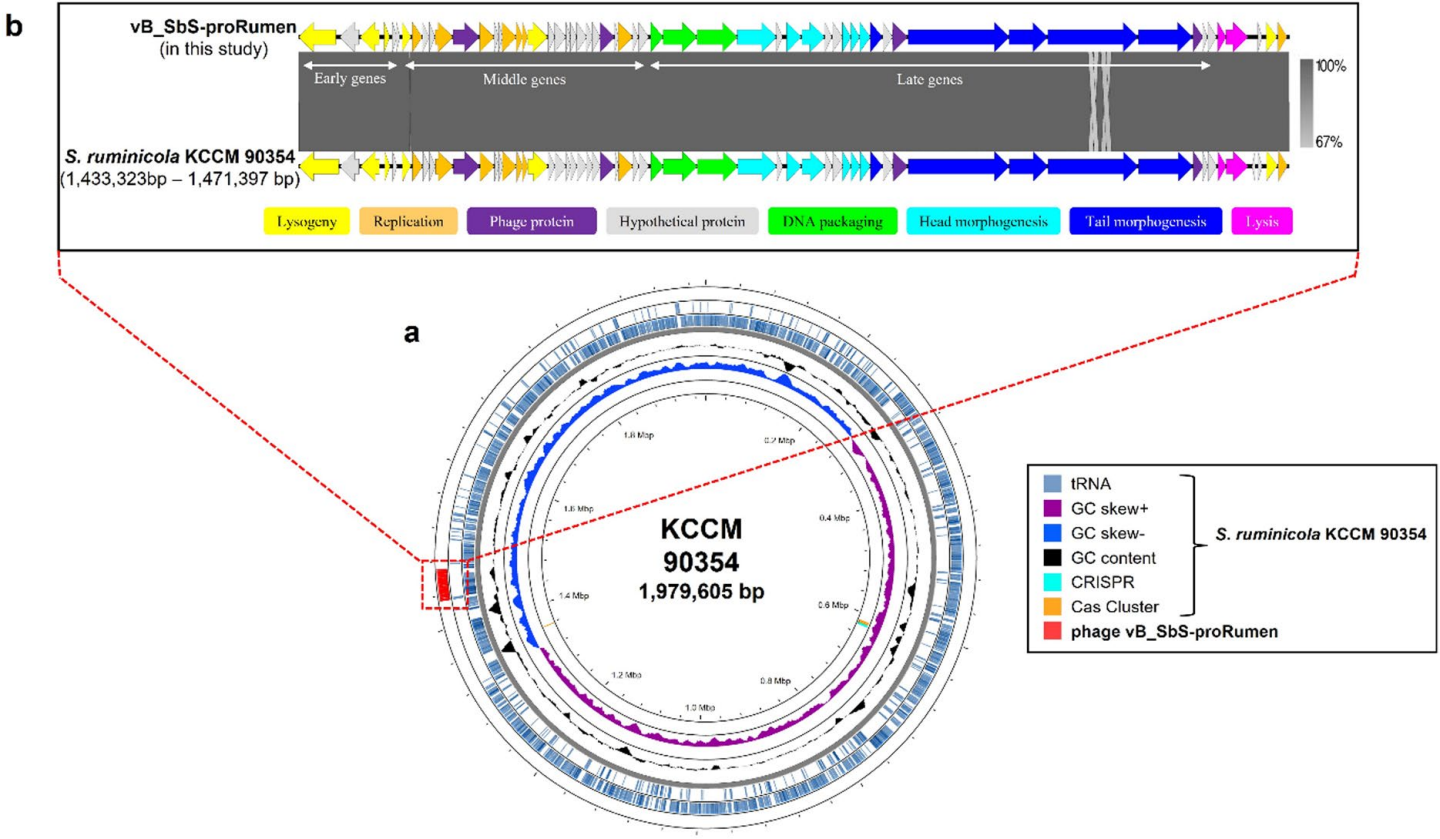
Integrated genomic region of the SBSEC temperate phage vB_SbS-proRumen into the SBSEC lysogenic strain KCCM 90354 chromosome. (**a**) Circular genome map of lysogeny generated using CGView; the contents of the feature rings and arrows are shown in different colors. In the chromosome of the prophage-carrying strain, the putative prophage region is presumed to be located from 1,433,323 to 1,471,397 bp (red). (**b**) Two linear genome maps showing the eight functional ORF clusters in different colors, constructed using EasyFig. Gray shading indicates nucleotide sequence identity (67–100%) between vB_SbS-proRumen and strain KCCM 90354. All ORFs shared 100% identity, while the lower identity values correspond to intergenic regions.

The prophage region in the chromosome (1,979,605 bp) of the lysogen was confirmed at positions 1,433,323–1,471,397 bp, corresponding to the 38,074 bp genome of vB_SbS-proRumen (Fig. 8a). A gene-level comparison of the two matching sequences, starting from the integrase region of vB_SbS-proRumen (ORF 1), revealed consistent arrangement and 100% BLASTn identity between corresponding ORFs, although sequence variations were detected in intergenic regions (Fig. 8b). The annotated genes of vB_SbS-proRumen, based on their putative functions and genomic arrangement, during the phage life cycle post-host infection, the genes of vB_SbS-proRumen were clustered as early (non-structural), middle (replication), or late (structural).

##### VIRIDIC and orthoANI-based intergenomic similarity analyses

**Fig. 9:**
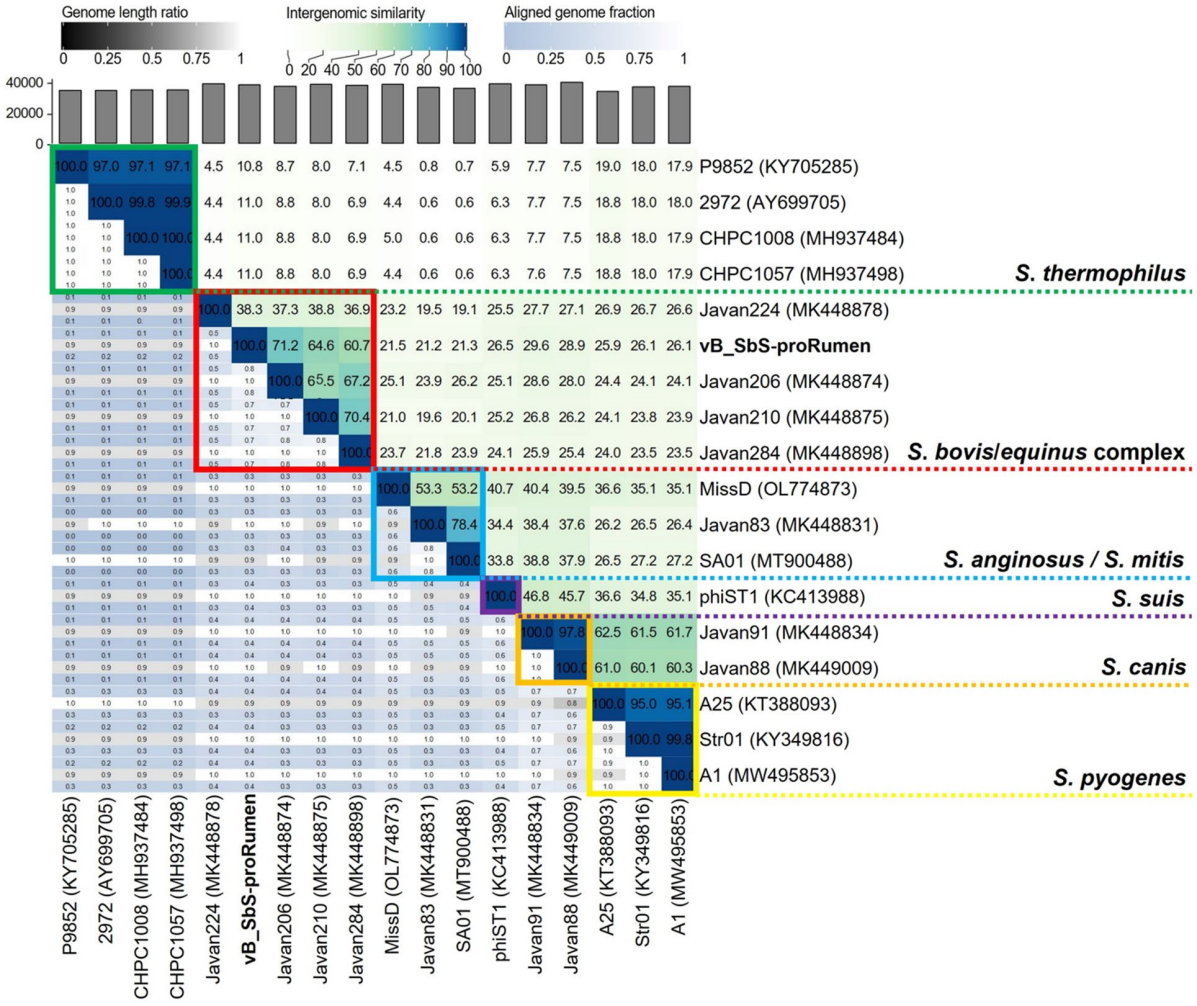
Nucleotide-based intergenomic similarities of SBSEC temperate phage vB_SbS-proRumen with 17 *Streptococcus* phages, including the four putative SBSEC prophage regions, using VIRIDIC. Top right: nucleotide similarity at a value of 0–100% between the genomes of each pair compared; darker colors represent genomes more closely related to phage vB_SbS-proRumen; Bottom left: aligned genome fraction and the genome length ratio at each value of 0–1.

Comparative genomic analysis was conducted between vB_SbS-proRumen and representative *Streptococcus* phages using heatmaps generated by orthoANI (**Fig. S7**) and the Virus Intergenomic Distance Calculator (VIRIDIC) (Fig. 9). Both analyses consistently showed clustering of the phage with the four putative SBSEC prophage regions, indicating a similarity range of 75.5–91.8% in the calculated ANI values and 38.3–71.2% in nucleotide-based intergenomic similarity.

##### Comparative genomic analysis with Streptococcus phages

**Fig. 10:**
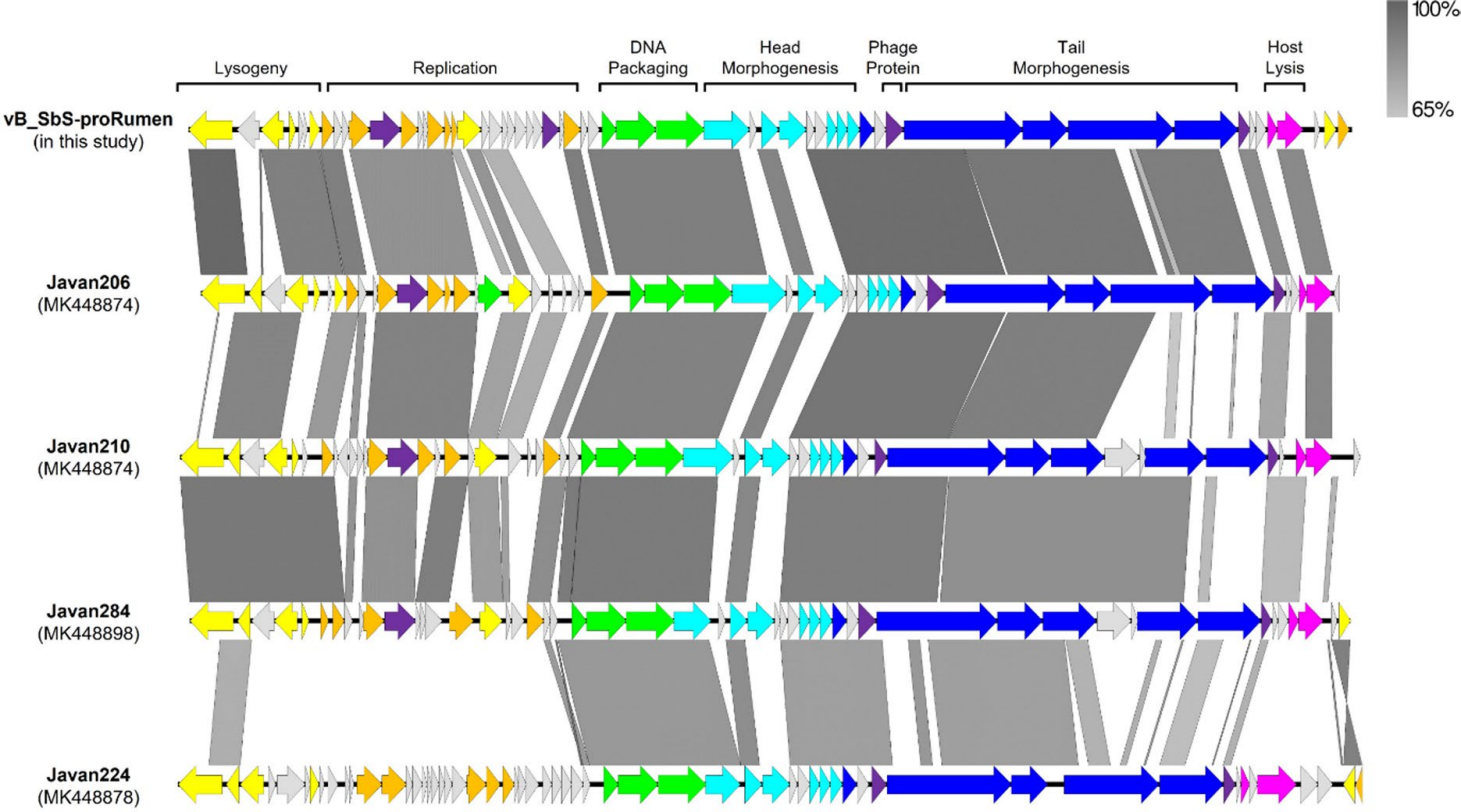
Comparison of the genome between SBSEC temperate phage vB_SbS-proRumen and predicted genomic regions in four putative SBSEC prophages, including Javan 210 (MK448875), Javan 206, (MK448874), Javan 284 (MK448898), and Javan 224 (MK448878), available in the GenBank database. The linear genome maps are constructed with arrows in eight different colors according to different functional ORFs using EasyFig. The gray regions indicate nucleotide sequence identity from 65 to 100%.

The genomic relationship between vB_SbS-proRumen and five related *Streptococcus* phages, including the four SBSEC prophage regions, was assessed using multiple approaches. A synteny map revealed highly similar connections between some vB_SbS-proRumen genes and those of other SBSEC prophage regions (**Fig. S6a**). The gene distribution analysis compared the genomic content of vB_SbS-proRumen with the predicted genomic regions of other SBSEC prophage regions, highlighting protein-coding genes exclusive to vB_SbS-proRumen and those shared with the other prophage regions. vB_SbS-proRumen possessed only 32 genes (55%) unique to its genome and absent in the compared *Streptococcus* phages, whereas the remaining genes were shared, ranging from one to eight genes per phage (**Fig. S6b**). In particular, *Streptococcus* prophage Javan 210 exhibited the most shared genes with vB_SbS-proRumen (**Table S6**). Among the shared genes, a single-stranded DNA-binding protein (SSB) was identified in the predicted genomic regions of representative SBSEC prophages, suggesting a potentially conserved role in the replication and maintenance of prophages within SBSEC strains. **Fig. S6c** presents the genetic similarities and differences between vB_SbS-proRumen and its related *Streptococcus* phages by illustrating the number of overlapping and unique genes for each phage. Additionally, comparative genomic analysis between vB_SbS-proRumen and the predicted genomic regions of four SBSEC prophages revealed a highly conserved genomic organization and overall similarity in gene arrangements based on the predicted functional genes (Fig. 10). While the overall structural organization was conserved, minor variations in gene content (e.g., ORF1 and ORF 2) were observed among the compared prophage regions.

## Discussion

SBSEC, including the newly proposed species, *S*. *ruminicola*, are opportunistic pathogens that cause ruminal lactic acidosis and are commonly found in the gastrointestinal tracts of humans and animals^6^. Recently, lytic phages infecting SBSEC were isolated and characterized, indicating their potential as novel biocontrol agents^25^. Previous genomic studies have confirmed the presence of putative prophages in SBSEC lysogens originating from ruminants^22,23^; however, research on the induction of these prophages and their potential as biocontrol agents remains limited. The present study is the first to report the characterization of an inducible prophage, vB_SbS-proRumen, harbored by *S*. *ruminicola*, a ruminant-derived SBSEC strain. In fact, a significant proportion of SBSEC strains harbor potentially inducible prophages, with 34.4% of the tested strains exhibiting either complete or partial lysis upon MMC treatment, consistent with previous reports^23^.

Strong prophage inducibility was observed in *S*. *ruminicola* KCCM 90354 following exposure to MMC, as evidenced by a pronounced decrease in OD_600_ across various MMC concentrations. The successful induction of vB_SbS-proRumen from its lysogenic host strain KCCM 90354 was demonstrated by a reduction in the OD_600_ of MMC-treated cultures and by the detection of infectious phage particles in the cell-free supernatant. The formation of clear plaques on the indicator host *S*. *ruminicola* KCCM 90384 confirms its suitability for propagating the phage. In contrast, the appearance of turbid plaques for several other strains suggests variable lytic activity by the phage or potential host defense mechanisms that impede efficient propagation. Such defenses may include receptor mutations decreasing phage adsorption efficiency, as well as intracellular systems, such as restriction-modification, abortive infection pathways, or CRISPR-Cas systems, all of which can restrict phage replication within the host cell^28,29^.

The host range analysis of SBSEC temperate phage vB_SbS-proRumen revealed its ability to infect multiple SBSEC strains and certain lactic acid bacteria, as validated by EOP assays. Relatively high EOP values (0.64–0.81) in strains with clear plaques and moderate EOP values (0.26–0.57) in those with turbid plaques indicate productive phage infection rather than residual mitomycin C or free endolysin activity. The observed cross-species infectivity suggests that the phage may be a viable candidate for targeting a broader spectrum of ruminal lactic acid bacteria implicated in ruminal lactic acidosis^30^. Nonetheless, as a temperate phage, its propensity for lysogeny rather than host lysis could limit its direct utility as a biocontrol agent. Thus, genetic engineering approaches, such as deleting integrase or repressor genes to prevent lysogeny, would be necessary before considering this phage for practical biocontrol applications^31^. The vB_SbS-proRumen, which belongs to the Siphovirus family, exhibited a morphotype commonly observed among lysogenic phages^32^. The results of the bacteriolytic activity of the phage against its indicator host strain, KCCM 90384, revealed effective inhibition of bacterial growth, particularly at the highest MOI. Furthermore, the rapid adsorption rate of vB_SbS-proRumen exhibited a strong affinity between the phage and its indicator host. The one-step growth curve of the phage revealed a relatively short lytic cycle and high progeny production, which are advantageous for rapid proliferation and dissemination in the rumen environment. Furthermore, the phage demonstrated stability over a wide pH range and at temperatures up to 37 °C. This is essential for its survival and effectiveness within the rumen, where ruminal acidosis often leads to a pH below 5.0 and temperatures of 38–40 °C^33^. However, given the significant decrease in phage viability between 37 and 45 °C, testing at intermediate temperatures (e.g., 40–42 °C) is warranted to more precisely determine the thermal limits of phage stability under physiologically relevant ruminal conditions. Moreover, the phage significantly inhibited biofilm formation and reduced pre-formed mature biofilm biomass in an MOI-dependent manner. This is particularly relevant in the rumen, where biofilm formation by ruminal bacteria may be a potential pathogenic factor in ruminants^34^.

The genomic analysis of the SBSEC lysogenic strain KCCM 90354 revealed that the VFs were primarily those commonly found in *Streptococcus* species^35^, suggesting that these factors may not necessarily confer enhanced pathogenicity to the strain. Furthermore, the identification of a Type II-A CRISPR-Cas system suggests a potential defense mechanism against foreign genetic elements; however, this system is not unique to non-pathogenic bacteria. Notably, none of the 25 CRISPR spacers corresponded to the prophage region of vB_SbS-proRumen, suggesting a lack of CRISPR-mediated immunity against this phage, which may have contributed to its successful integration into the host genome. The identification of the confirmed prophage region in the lysogen genome (1,433,323–1,471,397 bp) indicates the potential for horizontal gene transfer among rumen bacteria^36^. Prophages can act as vectors for disseminating genes encoding VFs, AMR, and other adaptive traits^37^, contributing to the adaptive ability conferred by lysogens in the rumen environment. This potential for horizontal gene transfer is a critical consideration in assessing temperate phages for biocontrol applications, as it may inadvertently promote the dissemination of undesirable genetic elements among bacteria.

The genome of vB_SbS-proRumen was estimated to be 38,092 bp, consistent with the typical range for Siphovirus phages infecting other-related lactic acid bacteria, which generally have 30–50 kb genomes and 35–40% GC contents^38^. The absence of VFs and ARGs in the phage genome, consistent with findings from lysogenomic analysis, is a favorable trait that underscores its suitability as a biocontrol candidate. However, this factor alone does not fully mitigate the risks associated with temperate phages, such as lysogenic conversion or the potential to acquire genes via recombination events. Functional annotation of the 58 putative ORFs in the phage genome revealed a modular gene arrangement. This characteristic of temperate phages allows efficient regulation of gene expression during lytic and lysogenic cycles^17^. The gene clusters were classified according to their transcription activity throughout the phage life cycle, consistent with typical gene arrangements found in predicted genomic regions of other SBSEC prophages (Fig. 10), and demonstrating the temporal regulation of phage gene expression^39^. The genome encodes six lysogen-associated proteins, including integrase (ORF 1), repressor proteins (ORFs 3, 4, 7, and 57), and anti-repressor proteins (ORF 19), each with crucial roles in determining the life cycle of the prophage after host infection. Additionally, two lytic-associated proteins, holin (ORF 54) and lysin (ORF 55), coordinate host cell lysis and phage progeny release. Furthermore, phylogenetic analysis of vB_SbS-proRumen showed that it clustered with several previously predicted SBSEC prophage regions. Moreover, it correlated with the phylogeny of its bacterial host as lysogenic *S*. *ruminicola* KCCM 90354 clustered with other SBSEC-type strains. This further supported the co-evolution of phages and their hosts in the rumen environment.

Genomic analysis of lysogenic *S*. *ruminicola* KCCM 90354 and its induced temperate phage vB_SbS-proRumen confirmed the integration of the prophage region (1,433,323–1,471,397 bp) into the bacterial chromosome, providing strong evidence for the lysogenic life cycle of the phage. The gene clusters were categorized according to their predicted functions and spatial arrangement, consistent with the conventional temporal gene organization observed in analogous genome regions of other SBSEC prophages^39^. Comparative genomic analyses using orthoANI and VIRIDIC confirmed that the phage shared a close taxonomic relationship with other putative SBSEC prophage regions, particularly Javan 210, 206, 284, and 224. Moreover, the comparative genome analysis of vB_SbS-proRumen with its related phages revealed unique genes (**Fig. S6**), suggesting the acquisition of novel genetic material during its evolutionary history^15^. However, those representative SBSEC prophage regions shared genes encoding essential functions, such as DNA replication, phage structure, and host lysis, with the common presence of the SSB gene^40^, indicating a conserved role in replicating and maintaining prophages within SBSEC strains. The function-based genomic comparison of vB_SbS-proRumen with the predicted genomic regions of four SBSEC prophages revealed considerable differences in the proteins that regulate lysogeny and host lysis. The unique gene arrangement between ORFs 1 and 2 in the phage suggests specialized lysogen regulation. These findings provide insights into functional diversification among closely related phages, highlighting the importance of comprehensive genomic comparisons in understanding phage–host interactions.

## Conclusions

In this study, a temperate phage, vB_SbS-proRumen, was successfully induced from SBSEC strains originating from ruminants, and its biological and genomic properties were characterized. Genomic analysis confirmed the integration of the prophage region into the bacterial chromosome, providing insights into the previously unknown diversity of SBSEC phages. Although the application of temperate phages in biocontrol raises well-defined concerns, including the potential for lysogenization and horizontal gene transfer, the analysis of vB_SbS-proRumen offers valuable insights to advance phage-based strategies targeting SBSEC-related infections or disorders. Additionally, the identification of essential functional proteins—including lytic (lysin) and lysogenic (integrase and repressor) proteins—from vB_SbS-proRumen establishes a foundation for future genetic engineering approaches to convert this temperate phage into a strictly lytic variant. By developing modified vB_SbS-proRumen virions through genetic engineering, it may be possible to enhance their lytic capacity while reducing the risks associated with lysogeny, thereby contributing to safer dairy products and improved public health outcomes.

## Materials and methods

### Bacterial strains and culture conditions

A total of 65 bacterial strains were used in this study, including 51 SBSEC isolates (three *S*. *ruminicola*, 44 *S*. *equinus*, and four *S*. *lutetiensis*), nine SBSEC type strains (*S. ruminicola* KCTC 43308^T^, *S. equinus* ATCC 9812^T^, *S. bovis* ATCC 33317^T^, *S. infantarius* subsp. *infantarius* ATCC BAA-102^T^, *S. gallolyticus* subsp. *gallolyticus* CCUG 35224^T^, *S. gallolyticus* subsp. *pastuerianus* CCUG 46150^T^, *S. gallolyticus* subsp. *macedonicus* ATCC BAA-249^T^, *S. lutetiensis* CCUG 46149^T^, and *S. alactolyticus* ATCC 43077^T^), and five other gram-positive-type strains (Table 1**)**. All strains were cultured on tryptic soybean agar (TSA; Difco, BD Biosciences, Franklin Lakes, NJ, USA) at 37 °C for 24 h and stored at − 80 °C in tryptic soybean broth (TSB; Difco, BD Biosciences, Franklin Lakes, NJ, USA) supplemented with 10% glycerol. For phage propagation and biological characterization, TSA soft agar (0.7% agar) was used. The phage lysate was subsequently amplified using the indicator strain *S. ruminicola* KCCM 90384, employing both the double-layer agar technique with TSA soft agar (0.7% agar) and broth-based propagation as previously described^25^. This process achieved a final titer of approximately 5 × 10⁸ PFU/mL.

### Screening inducible prophage-possessing SBSEC strains

The presence of potential-inducible prophages in 65 bacterial strains, including 50 SBSEC isolates, nine SBSEC type strains, and five other available gram-positive-type strains (Table 1), was investigated using mitomycin C as an induction agent. Overnight bacterial cultures, prepared as previously described^9^, were diluted 1:100 in fresh TSB and incubated until the optical density at 600 nm (OD_600_) reached 0.05–0.2. At this point, MMC was added to the cultures to achieve a final concentration of 1 µg/mL (or 0.1 and 0.5 µg/mL for the confirmed lysogen). The OD_600_ of the inoculums was measured hourly for 5 h at 37 °C to monitor the growth reduction and lysis of the bacterial strains. The percent reduction in OD_600_ was calculated by comparing the OD_600_ values of MMC-treated cultures with those of untreated controls at 5 h. At the final time point, the cultures were centrifuged at 10,000 × *g* for 15 min, and the supernatants were passed through a 0.22-µm filter to remove unlysed bacterial cells and debris, yielding cell-free phage suspensions. The strains were categorized into three levels of lysis based on reduced OD_600_ values compared to the untreated control: complete lysis (> 80% reduction), partial lysis (60–80% reduction), and no lysis (< 60% reduction). The initial OD_600_ values of the cultures were measured before MMC treatment.

### Identifying a temperate phage-susceptible SBSEC strain and characterizing its host range

To identify the temperate phage-susceptible SBSEC strain (indicator host) and determine the host range of the induced phage, the undiluted phage-suspected suspensions (approximately 5 × 10^8^ PFU/mL) were tested against a panel of 65 strains using the double-layer agar method^25^. Plaques were formed and categorized based on their appearance. Clear plaques indicated complete bacterial lysis, while turbid plaques suggested partial lysis or growth inhibition by other cellular components. The SBSEC strain exhibiting the highest susceptibility to the induced phage, as evidenced by the formation of clear plaques, was selected as the indicator host for further phage amplification and characterization.

### Biological characteristics of SBSEC Phage vB_SbS-proRumen

#### Transmission electron microscopy

The morphology of the SBSEC temperate phage was examined using TEM. Briefly, a 10-µL aliquot of the phage suspension (ca. 10^8^ PFU/mL) was spotted onto a 200-mesh nickel grid (Ted Pella, Inc., Redding, CA, USA), negatively stained with 5% uranyl acetate, washed with distilled water, and air-dried. The grid was observed using a transmission electron microscope (JEM-1011; JEOL, Tokyo, Japan) at an acceleration voltage of 80 kV.

#### Bacteriolytic activity

The antibacterial activity of the SBSEC temperate phage was determined using *S*. *ruminicola* KCCM 90384 as an indicator host, as previously described^25^. The phage suspension was obtained by propagating vB_SbS-proRumen on the indicator strain KCCM 90384 without mitomycin C. Briefly, a 5-mL aliquot of freshly grown host bacterial cells (ca. 10^7^ colony-forming unit (CFU)/mL) was mixed with 5 mL of the phage suspension at four different MOIs ranging from 0.001 to 1. Thereafter, the mixtures were incubated at 37 °C with shaking, and the OD_600_ was measured hourly for 9 h using a spectrophotometer (OPTIZEN POP; Klab Inc., Daejeon, Korea). The control was bacterial cultures without phages. All experiments were performed in triplicate.

#### Adsorption rate and one-step growth curve

The optimal adsorption time and one-step growth curve of the SBSEC temperate phage were determined using *S*. *ruminicola* KCCM 90384 as the indicator host, as previously described^25^. For the adsorption assay, 1 mL of early-exponential-phase host bacterial cells (ca. 10^7^ CFU/mL) was mixed with the phage suspension (ca. 10^5^ PFU/mL) to achieve an MOI of 0.01, and the mixture was incubated at 37 °C. Next, the samples were collected 0, 5, 10, 15, 20, 25, and 30 min post-inoculation and centrifuged at 10,000 × *g* for 5 min to remove bacterial cells and adsorbed phages; the titer of non-adsorbed phages in the supernatants was determined using the double-layer agar method. For the one-step growth curve, 10 mL of early-exponential-phase host bacterial cells (ca. 10^7^ CFU/mL) was infected with the phage suspension at an MOI of 0.01 and incubated at 37 °C for 10 min, allowing over 80% of the phages to adsorb onto the host cells. The culture was centrifuged at 12,000 × *g* for 5 min to remove non-adsorbed phages, and the pellet was resuspended in pre-heated TSB before incubation at 37 °C with shaking. Subsequently, the samples were collected at 0, 10, 20, 30, 40, 50, 60, 70, 80, and 90 min, and phage titers were determined using the double-layer agar method. Burst size was calculated by dividing the final phage titer measured after the burst period by the initial count of infected cells at the start of the latent phase:

$$\begin{aligned} & {\text{Burst size}} = ({\text{final PFU}}/{\mathrm{mL}} - {\text{initial PFU}}/{\mathrm{mL}}) \\ & \quad /{\text{initial number of infected cells }}\left( {{\mathrm{PFU}}/{\text{mL at time }}0} \right). \\ \end{aligned}$$

All experiments were conducted in triplicate.

#### pH and thermal stability

The pH and thermal stability of the SBSEC temperate phage were assessed using *S*. *ruminicola* KCCM 90384 as an indicator host, as previously described^25^. For pH stability, a phage suspension (ca. 10^7^ PFU/mL) was tested under 11 pH conditions (2, 3, 4, 4.6, 5, 6, 7, 8, 9, 10, and 11) at 37 °C for 3 h. The phage titer was determined, and the resulting values were compared with those of a control maintained at pH 7. For thermal stability, a phage suspension (ca. 10^7^ PFU/mL) was incubated at seven temperatures (4, 25, 30, 37, 45, 56, and 80 °C) for 3 h. The phage titer was determined, and the resulting values were compared with those of a control maintained at 4 °C. All experiments were performed in triplicate.

#### Anti-biofilm activity

The anti-biofilm activity of the SBSEC temperate phage was evaluated using two approaches: (i) prevention of biofilm formation and (ii) degradation of pre-formed biofilms. The experiments were conducted at MOIs of 0.001, 0.01, 0.1, 1, and 10, as previously described^25^.

For the biofilm prevention assay, overnight-cultured *S*. *ruminicola* KCCM 90384 (ca. 10^8^ CFU/mL) was diluted 1:100 in fresh TSB supplemented with 1% glucose (TSBG). A 100-µL aliquot of the diluted bacterial suspension was added to each well of a flat-bottomed 96-well plate (SPL Life Sciences, Pocheon, Korea), followed by the addition of 100 µL of phage lysate at different MOIs prepared in phosphate-buffered saline (PBS; pH 7.5; Thermo Fisher Scientific, Waltham, MA, USA). The control well contained only the bacterial suspension without phages. Subsequently, the plates were incubated at 37 °C for 24 h without shaking. After incubation, each well was washed three times with PBS to remove the remaining planktonic cells. Next, the biofilms were stained with 200 µL of 0.1% CV (Sigma-Aldrich) for 20 min at 25 °C. The bound CV was solubilized with 100 µL 95% ethanol, and the total biofilm mass was quantified by measuring the OD_600_ with a microplate reader (Spectramax 190; Molecular Devices, San Jose, CA, USA). For the biofilm degradation assay, 200 µL of bacterial culture diluted in fresh TSBG (ca. 10^6^ CFU/mL) was added to each well of a 96-well plate and incubated at 37 °C for 24 h to allow mature biofilm formation. After incubation, each well was washed three times with PBS to remove planktonic cells. Thereafter, 200 µL of phage lysate at different MOIs was added to the wells containing the pre-formed biofilms. The control wells contained PBS without phages. After 24 h of incubation at 37 °C, the supernatant was removed, and the remaining biofilms were stained with 0.1% CV as described above. Finally, biofilm mass was determined by measuring the OD_600_ using a microplate reader. All experiments were performed in triplicate.

### Genomic characteristics of SBSEC phage vB_SbS-proRumen

#### Whole-genome sequencing

To compare the genetic characteristics of the integrated prophage in the SBSEC genome with those of the amplified temperate phage, two sequencing approaches were used. First, the genomic DNA (gDNA) of a prophage-inducible SBSEC strain, *S*. *ruminicola* KCCM 90354, was extracted using a DNeasy Blood & Tissue kit (Qiagen, Hilden, Germany). The extracted gDNA was sequenced using both long-read and short-read platforms. Long-read sequencing was performed on a MinION Flow Cell R10.4.1 (Oxford Nanopore Technologies, Oxford, UK), while short-read sequencing was conducted on an Illumina HiSeq X-10 platform (Illumina, San Diego, CA, USA). For Nanopore sequencing, libraries were prepared with the Native Barcoding Kit 24 V14 SQK-NBD 114.24 (Oxford Nanopore Technologies), and for Illumina libraries, the TruSeq Nano DNA library prep kit was used. After sequencing, Nanopore raw reads (1,586,402,097 bp; 238,701 reads) were filtered for quality using NanoFilt v2.8.0 (Q ≥ 15; length ≥ 5,000 bp), resulting in 32,311 high-quality reads and a total of 421,690,302 bp. Illumina paired-end raw reads (1,984,565,000 bp; 13,142,814 reads) were filtered with Fastp v1.0.1 (Q ≥ 30), yielding 10,713,636 high-quality reads totaling 1,617,611,000 bp. The hybrid genome assembly was performed with Unicycler v0.5.1, which combines short and long reads to accurately reconstruct bacterial genomes. Second, the gDNA of the SBSEC temperate phage suspension (ca. 10^9^ PFU/mL), amplified using an SBSEC indicator host, *S*. *ruminicola* KCCM 90384, was extracted using the phenol-chloroform method, as previously described^41^. For DNA library construction, gDNA was sequenced on an Illumina HiSeq X-10 platform (Illumina) by Macrogen (Seoul, Korea), yielding 151-bp paired-end reads. Raw reads were assessed for quality using FastQC v. 0.11.5^42^ and trimmed using Trimmomatic v. 0.36^43^. *De novo* genome assembly was performed using SPAdes v. 3.13.0^44^ with default parameters, and the resulting assembly was polished.

#### Genomic analysis

The annotated genome of a prophage-inducible SBSEC strain, *S*. *ruminicola* KCCM 90354, was analyzed for the presence of VFs, ARGs, MGEs, CRISPR-Cas system, and prophage regions using online databases, including the Virulence Factors Database (VFDB)^45^, Antibiotic Resistance Gene-ANNOTation (ARG-ANNOT)^46^, Mobile Element Finder^47^, CRISPRCasFinder^48^, and PHAge Search Tool Enhanced Release (PHASTER)^49^, respectively. Genome-based phylogenetic analysis of *S*. *ruminicola* KCCM 90354 was performed using TYGS^50^ with several strains, including six other *Streptococcus* species (SBSEC, *S*. *anginosus*, *S*. *mitis*, *S*. *canis*, *S*. *pyogenes*, and *S*. *thermophilus*) obtained from the NCBI GenBank database. orthoANI values were calculated using the OAT tool^51^ to analyze the genomic relatedness of SBSEC type strains. A circular genome map was constructed using the CGView server^52^ and PATRIC^53^.

The complete genome of the SBSEC temperate phage was initially annotated using Rapid Annotation Subsystem Technology (RAST)^54^, and the functional annotation of the ORFs was predicted using BLASTp^55^, Pfam^56^, and HHpred^57^. Each predicted ORF was analyzed for pI Mw, transmembrane domain (TMHMM), and signal peptides using online database tools, including Compute pI/Mw^58^, TMHMM-2.0^59^, and SignalP-5.0^60^. The presence of tRNA genes, VFs, and ARGs in the SBSEC phage genome was screened using tRNAscan-SE-2.0^61^, CARD, and ARG-ANNOT. Phage termini were identified using PhageTerm^62^. The whole genome phylogeny of the SBSEC phage was constructed using VICTOR^63^. Intergenomic comparison and relatedness of the induced SBSEC phage to other *Streptococcus* phages were assessed using VIRIDIC^64^ and EasyFig^65^, with results plotted using the R package “heatmap” (R Foundation for Statistical Computing, Vienna, Austria)^66^. Comparative genomic synteny was illustrated using Circoletto^67^, and the genome map was visualized using the CGView server. Gene phylogeny based on the terminase large subunit (TerL) was performed using the maximum-likelihood method in MEGA X^68^. InteractiVenn^69^ and DiVenn^70^ were used to generate Venn diagrams illustrating the distribution of genes annotated by the UniProtKB/Swiss-Prot database^71^ from the SBSEC phage across five related SBSEC phages.

### Statistical analysis

All experiments were conducted independently in triplicate, with data presented as mean ± standard deviation (SD). Statistical analysis was performed using GraphPad Prism 7 software (GraphPad Software, La Jolla, CA, USA), employing one-way analysis of variance (ANOVA) to determine significant differences between groups, with a *p*-value threshold of 0.05. To identify specific group differences, *post hoc* analyses of significant ANOVA results were conducted using Dunnett’s multiple-comparison test to compare experimental conditions with their respective controls.

## Supplementary Information

Below is the link to the electronic supplementary material.

Supplementary Material 1

Supplementary Material 2

## Data Availability

The SBSEC temperate phage vB_SbS-proRumen was deposited in the Korean Collection for Type Culture (KCTC) under KCTC 15920BP. The complete genome sequence of the SBSEC temperate phage vB_SbS-proRumen was deposited in GenBank under the accession number PP793892. The complete genome sequence of S. ruminicola KCCM 90354 was deposited in DDBJ/EMBL/GenBank under the accession number CP156694. The NCBI BioProject accession number is PRJNA1105708, and the BioSample accession number is SAMN41108009.
