# Supplementary material for: Characterization of a temperate bacteriophage isolated from the ruminant-derived Streptococcus bovis/equinus complex
Source: Sci Rep. 2026 May 17;16:22338. doi: 10.1038/s41598-026-50797-4 (PMC13376510; doi:10.1038/s41598-026-50797-4)
Supplement: Supplementary file 2 — Supplementary Material 2 [file 41598_2026_50797_MOESM2_ESM.docx]

Characterization of a temperate bacteriophage isolated from the ruminant-derived *Streptococcus bovis/equinus* complex

Seon Young Park^1,2,6^, Jun-Seob Kim^3,6^, Do Young Jin^3^, Ye Bin Kim^4^, Tae Seon Cha^4^, Soojin Lim^5^, Seunghui Lee^4^, Seongwon Seo^2,*^, and Ji Hyung Kim^4,*^

^1^ Veterinary Drugs and Biologics Division, Animal and Plant Quarantine Agency, Gimcheon, 39660, Republic of Korea; [lovesun139@korea.kr](mailto:lovesun139@snu.ac.kr)

^2^ Division of Animal and Dairy Sciences, College of Agriculture and Life Science, Chungnam National University, Daejeon 34134, Republic of Korea; [swseo@cnu.ac.kr](mailto:swseo@cnu.ac.kr)

^3^ Department of Nano-Bioengineering, Incheon National University, Incheon, 22012, Republic of Korea; [junkim@inu.ac.kr](mailto:junkim@inu.ac.kr) (J.K.); [ehdud0505@inu.ac.kr](mailto:ehdud0505@inu.ac.kr) (D.Y.J.)

^4^ Department of Food Science and Biotechnology, College of Bionano Technology, Gachon University, Seongnam 13120, Republic of Korea; [zxc5620@gachon.ac.kr](mailto:zxc5620@gachon.ac.kr) (Y.B.K); [walnut99@gachon.ac.kr](mailto:walnut99@gachon.ac.kr) (T.S.C.); [seunghi@gachon.ac.kr](mailto:seunghi@gachon.ac.kr) (S.L.); [kzh81@gachon.ac.kr](mailto:kzh81@gachon.ac.kr) (J.H.K.)

^5^ Laboratory of Aquatic Biomedicine, College of Veterinary Medicine and Research Institute for Veterinary Science, Seoul National University, Seoul 08826, Republic of Korea; [sjlim001103@snu.ac.kr](mailto:sjlim001103@snu.ac.kr)

^6^ These authors contributed equally: Seon Young Park and Jun-Seob Kim.

*****Correspondence: [swseo@cnu.ac.kr](mailto:swseo@cnu.ac.kr) (S.S.), [kzh81@gachon.ac.kr](mailto:kzh81@gachon.ac.kr) (J.H.K.), Tel.: +82-31-750-5383; Fax: +82-31-750-5389

**Supplementary tables**

**Table S2.** Prediction of prophage region in *S*. *ruminicola* KCCM 90354 using PHASTER.

| **No.** | **Location** | **BLAST Hit** | | **E-value** |
| --- | --- | --- | --- | --- |
|  |  | **Putative function** | **Match organism (accession no.)** |  |
| 1 | 1433081..1433092 | attL | - | - |
| 2 | 1433244..1433345 | hypothetical protein | *Streptococcus equinus* strain MDC1 (CP059471.1) | 0.007 |
| 3 | 1433323..1434759 | phage integrase protein | phage_Strept_DCC1738 (NC_024361) | 0 |
| 4 | 1434902..1435642 | putative protease | phage_Strept_phiNJ2 (NC_019418) | 3.14e-07 |
| 5 | 1435691..1436425 | putative repressor | phage_Strept_SMP (NC_008721) | 4.02e-108 |
| 6 | 1436619..1436825 | hypothetical protein | phage_Strept_SMP (NC_008721) | 1.16e-23 |
| 7 | 1437081..1437224 | hypothetical protein | phage_Strept_phiARI0031 (NC_031910) | 3.94e-09 |
| 8 | 1437321..1437575 | putative replication protein | phage_Strept_315.4 (NC_004587) | 1.75e-28 |
| 9 | 1437699..1438079 | hypothetical protein | phage_Strept_315.3 (NC_004586) | 2.66e-18 |
| 10 | 1438089..1438346 | hypothetical protein | phage_Strept_IC1 (NC_024370) | 5.27e-09 |
| 11 | 1438346..1438570 | hypothetical protein | phage_Strept_315.1 (NC_004584) | 9.71e-19 |
| 12 | 1438581..1439258 | hypothetical protein | phage_Strept_phiNJ2 (NC_019418) | 1.35e-67 |
| 13 | 1439270..1440277 | hypothetical protein | phage_Strept_MM1 (NC_003050) | 1.95e-118 |
| 14 | 1440291..1440842 | putative mazG nucleotide pyrophosphohydrolase | phage_Strept_phiNJ2 (NC_019418) | 4.29e-34 |
| 15 | 1440847..1441014 | hypothetical protein | phage_Strept_phiARI0746 (NC_031907) | 9.19e-16 |
| 16 | 1441011..1441169 | hypothetical protein | *Streptococcus equinus* strain MDC1 (CP059471.1) | 2e-25 |
| 17 | 1441153..1441698 | phage ssDNA binding protein | phage_Strept_phiBHN167 (NC_022791) | 5.74e-09 |
| 18 | 1441708..1441935 | hypothetical protein | phage_Strept_phiNJ2 (NC_019418) | 1.13e-11 |
| 19 | 1441932..1442135 | hypothetical protein | phage_Strept_phiARI0746 (NC_031907) | 6.44e-15 |
| 20 | 1442132..1442905 | putative anti-epressor | phage_Strept_phi3396 (NC_009018) | 6.29e-95 |
| 21 | 1442919..1443167 | hypothetical protein | *Streptococcus* phage Javan210 (MK448875.1) | 3e-32 |
| 22 | 1443167..1443601 | hypothetical protein | *Streptococcus* phage Javan210 (MK448875.1) | 6e-73 |
| 23 | 1443601..1443777 | hypothetical protein | *Streptococcus* phage Javan210 (MK448875.1) | 7e-28 |
| 24 | 1443767..1444012 | hypothetical protein | *Streptococcus* phage Javan215 (MK448705.1) | 4e-33 |
| 25 | 1444016..1444399 | hypothetical protein | *Streptococcus* phage Javan215 (MK448705.1) | 2e-56 |
| 26 | 1444386..1444622 | hypothetical protein | *Streptococcus lutetiensis* NCTC8738 (LS483348.1) | 5e-35 |
| 27 | 1444612..1444917 | hypothetical protein | - | - |
| 28 | 1444914..1445477 | hypothetical protein | phage_Strept_315.1 (NC_004584) | 9.42e-30 |
| 29 | 1445474..1445620 | hypothetical protein | *Caudoviricetes* sp. (BK020608.1) | 7e-52 |
| 30 | 1445617..1446171 | putative endodeoxyribonuclease rusA | phage_Strept_A25 (NC_028697) | 5.67e-49 |
| 31 | 1446164..1446379 | hypothetical protein | *Streptococcus* phage Javan199 (MK448702.1) | 6e-11 |
| 32 | 1446399..1446779 | hypothetical protein | phage_Strept_phiNJ2 (NC_019418) | 1.29e-54 |
| 33 | 1446861..1447343 | putative terminase small subunit | phage_Strept_A25 (NC_028697) | 9.84e-68 |
| 34 | 1447333..1448643 | putative phage terminase large subunit | phage_Strept_phiNJ2 (NC_019418) | 0 |
| 35 | 1448653..1450203 | portal protein | phage_Strept_A25 (NC_028697) | 0 |
| 36 | 1450196..1451698 | putative phage minor capsid protein 2 | phage_Strept_phiNJ2 (NC_019418) | 0 |
| 37 | 1451695..1451925 | hypothetical protein | *Streptococcus* phage Javan210 (MK448875.1) | 2e-35 |
| 38 | 1452087..1452653 | putative scaffolding protein | phage_Strept_A25 (NC_028697) | 1.25e-96 |
| 39 | 1452668..1453555 | putative major capsid protein | phage_Strept_A25 (NC_028697) | 1.52e-157 |
| 40 | 1453557..1453817 | hypothetical protein | phage_Strept_A25 (NC_028697) | 3.65e-17 |
| 41 | 1453848..1454240 | putative phosphate-selective porin O and P | phage_Strept_phiNJ2 (NC_019418) | 2.2e-66 |
| 42 | 1454230..1454556 | putative minor capsid protein | phage_Strept_A25 (NC_028697) | 1.04e-40 |
| 43 | 1454556..1454903 | putative capsid protein | phage_Strept_A25 (NC_028697) | 3.25e-42 |
| 44 | 1454903..1455301 | putative minor capsid protein | phage_Strept_phiNJ2 (NC_019418) | 5.56e-71 |
| 45 | 1455312..1455770 | putative major tail shaft protein | phage_Strept_A25 (NC_028697) | 2.0e-59 |
| 46 | 1455784..1456161 | hypothetical protein | phage_Strept_A25 (NC_028697) | 1.34e-32 |
| 47 | 1456161..1456742 | hypothetical protein | phage_Strept_A25 (NC_028697) | 1.84e-90 |
| 48 | 1456757..1460641 | tail protein | phage_Strept_2972_NC_007019 | 0 |
| 49 | 1460638..1462125 | putative phage tail protein | phage_Strept_phiNJ2 (NC_019418) | 0 |
| 50 | 1462126..1465593 | phage capsid and scaffold protein | phage_Strept_20617 (NC_023503) | 0 |
| 51 | 1465610..1467694 | tail protein | phage_Strept_Abc2 (NC_013645) | 0 |
| 52 | 1467709..1468092 | hypothetical protein | phage_Strept_858 (NC_010353) | 1.25e-50 |
| 53 | 1468070..1468258 | hypothetical protein | phage_Strept_2972 (NC_007019) | 4.03e-23 |
| 54 | 1468279..1468611 | hypothetical protein | phage_Strept_2972 (NC_007019) | 8.61e-58 |
| 55 | 1468656..1468979 | holin | phage_Strept_2972 (NC_007019) | 9.75e-56 |
| 56 | 1468979..1469824 | phage lysin | phage_Strept_20617 (NC_023503) | 5.1e-139 |
| 57 | 1470525..1470911 | hypothetical protein | phage_Strept_PH15 (NC_010945) | 3.1e-31 |
| 58 | 1471077..1471088 | attR | - | - |

**Table S3.** Functional grouping of SBSEC temperate phage vB_SbS-proRumen predicted ORFs and their homology to other organisms.

| **Gene product** | | | | | | | | **Putative function**  **[Conserved domain]** | **Best match organism**  **(E-value)** | **Identity**  **(%)** | **Prediction** | |
| --- | --- | --- | --- | --- | --- | --- | --- | --- | --- | --- | --- | --- |
| **ORF** | **Strand** | **Start**  **(bp)** | **End**  **(bp)** | **Size**  **(aa)** | **MW**  **(kDa)** | **pI** | **Start**  **codon** |  |  |  | **TMHHM** | **SignalP** |
| 1 | - | 1 | 1437 | 478 | 55.4 | 9.2 | ATG | Recombinase family protein [PF00239; Resolvase; Resolvase, N terminal domain] | *S. lutetiensis* (0.0) | 99.6 | 0 | N |
| 2 | - | 1580 | 2320 | 246 | 27.4 | 9.3 | ATG | Hypothetical protein | *S. lutetiensis* (5e-172) | 96.3 | 2 | N |
| 3 | - | 2369 | 3103 | 244 | 28.2 | 4.6 | ATG | XRE family transcriptional regulator  [PF00717; Peptidase_S24; Peptidase S24-like] | *S. equinus* (3e-169) | 97.5 | 0 | N |
| 4 | + | 3297 | 3503 | 68 | 7.8 | 5.3 | ATG | Helix-turn-helix transcriptional regulator [IPR010982; Lambda_DNA-bd_dom_sf; Lambda repressor-like, DNA-binding domain superfamily] | *Streptococcus* sp. (4e-40) | 98.5 | 0 | N |
| 5 | + | 3610 | 3783 | 57 | 6.7 | 9.4 | ATG | Hypothetical protein | *S. gallolyticus* (1e-25) | 87.5 | 0 | N |
| 6 | + | 3759 | 3902 | 47 | 5.3 | 8.3 | ATG | Hypothetical protein | *S. lutetiensis* (4e-25) | 97.9 | 0 | N |
| 7 | + | 3999 | 4253 | 84 | 9.8 | 9.1 | TTG | Transcriptional regulator | *S. gallolyticus* (3e-50) | 92.9 | 0 | N |
| 8 | + | 4377 | 4757 | 126 | 14.6 | 4.6 | ATG | DnaD domain protein [PF07261; DnaB_2; Replication initiation and membrane attachment] | *S. equins* (7e-84) | 98.4 | 0 | N |
| 9 | + | 4767 | 5024 | 85 | 9.8 | 4.6 | ATG | Hypothetical protein | *S. lutetiensis* (1e-45) | 90.6 | 0 | N |
| 10 | + | 5024 | 5248 | 74 | 8.6 | 8.1 | ATG | Hypothetical protein | *S. equinus* (1e-43) | 93.2 | 0 | N |
| 11 | + | 5259 | 5936 | 225 | 25.8 | 6.2 | ATG | ERF family protein | *S. lutetiensis* (2e-139) | 90.7 | 0 | N |
| 12 | + | 5948 | 6955 | 335 | 38.7 | 4.8 | ATG | Phage protein  [IPR009785; Prophage_Lj928_Orf309; Lactobacillus prophage Lj928, Orf309] | *S. equinus* (0.0) | 97.9 | 0 | N |
| 13 | + | 6969 | 7520 | 183 | 20.4 | 5.4 | ATG | MazG-like family protein [PF03819; MazG; MazG nucleotide pyrophosphohydrolase domain] | *S. equinus* (1e-126) | 98.4 | 0 | N |
| 14 | + | 7525 | 7692 | 55 | 6.2 | 4.6 | ATG | Hypothetical protein | *S. infantarius* subsp.  *infantarius* (4e-30) | 100 | 0 | N |
| 15 | + | 7689 | 7847 | 52 | 6.0 | 5.5 | ATG | Hypothetical protein | *S. infantarius* subsp.  *infantarius* (1e-29) | 100 | 1 | N |
| 16 | + | 7831 | 8376 | 181 | 20.3 | 4.8 | ATG | Single-strand DNA-binding protein [IPR012340; NA-bd_OB-fold; Nucleic acid-binding, OB-fold] | *S. lutetiensis* (5e-119) | 96.7 | 0 | N |
| 17 | + | 8386 | 8613 | 75 | 9.0 | 9.6 | ATG | Hypothetical protein | *S. lutetiensis* (2e-44) | 97.3 | 0 | N |
| 18 | + | 8610 | 8813 | 67 | 8.0 | 9.5 | ATG | Hypothetical protein | *S. lutetiensis* (1e-40) | 97 | 1 | N |
| 19 | + | 8810 | 9583 | 257 | 29.5 | 8.3 | ATG | Phage antirepressor protein [IPR003497; BRO_N_domain; BRO N-terminal domain] | *S. lutetiensis* (5e-141) | 78.2 | 0 | N |
| 20 | + | 9597 | 9845 | 82 | 9.6 | 6.3 | ATG | Hypothetical protein | *S. infantarius* subsp.  *infantarius* (2e-50) | 95.1 | 1 | N |
| 21 | + | 9845 | 10279 | 144 | 17.1 | 9.7 | ATG | Hypothetical protein | *S. equinus* (3e-83) | 88.9 | 0 | N |
| 22 | + | 10279 | 10455 | 58 | 6.8 | 4.8 | ATG | Hypothetical protein | *S. equinus* (3e-32) | 96.6 | 0 | N |
| 23 | + | 10445 | 10690 | 81 | 9.1 | 6.8 | ATG | Hypothetical protein | *S. equinus* (4e-38) | 75.6 | 0 | Y |
| 24 | + | 10694 | 11077 | 127 | 14.6 | 4.8 | ATG | Hypothetical protein | *S. equinus* (5e-64) | 80.8 | 0 | N |
| 25 | + | 11064 | 11300 | 78 | 9.3 | 8.1 | ATG | Hypothetical protein | *S. equinus* (2e-42) | 92.2 | 0 | N |
| 26 | + | 11290 | 11595 | 101 | 11.8 | 6.2 | ATG | No match | - | - | 1 | N |
| 27 | + | 11592 | 12155 | 187 | 22.2 | 4.8 | ATG | Phage protein [IPR012865; DUF1642; Protein of unknown function DUF1642] | *S. vicugnae* (3e-71) | 61.7 | 0 | N |
| 28 | + | 12152 | 12298 | 48 | 5.5 | 4.1 | ATG | Hypothetical protein | *S. gallolyticus* (4e-14) | 70.5 | 0 | N |
| 29 | + | 12295 | 12849 | 184 | 21.3 | 9.3 | ATG | Phage protein [IPR036614; RusA-like_sf; Holliday junction resolvase RusA-like superfamily] | *S. lutetiensis* (9e-130) | 95.1 | 0 | N |
| 30 | + | 12842 | 13057 | 71 | 8.8 | 9.1 | ATG | Hypothetical protein | *S. equinus* (9e-42) | 95.8 | 0 | N |
| 31 | + | 13077 | 13457 | 126 | 15.2 | 9.6 | ATG | Hypothetical protein | *S. gallolyticus* (1e-71) | 84.1 | 0 | N |
| 32 | + | 13539 | 14021 | 160 | 18.2 | 5.1 | ATG | Phage terminase small subunit [IPR005335; Terminase_ssu; Terminase small subunit] | *S. equinus* (4e-109) | 99.4 | 0 | N |
| 33 | + | 14011 | 15321 | 436 | 50.3 | 8.7 | ATG | Phage terminase large subunit [IPR006437; Phage_terminase_lsu; Bacteriophage terminase, large subunit] | *S. equinus* (0.0) | 98.9 | 0 | N |
| 34 | + | 15331 | 16881 | 516 | 58.6 | 5.0 | ATG | Phage portal protein [IPR006432; Portal_putative_A118-type; Portal protein, putative, A118-type] | *S. equinus* (0.0) | 94.8 | 0 | N |
| 35 | + | 16874 | 18376 | 500 | 57.2 | 9.3 | ATG | Phage minor capsid protein [PF06152; Phage_min_cap2_Phage minor capsid protein 2] | *S. infantarius* subsp.  *infantarius* (0.0) | 97.2 | 0 | N |
| 36 | + | 18373 | 18603 | 76 | 8.9 | 4.2 | ATG | Hypothetical protein | *S. lutetiensis* (4e-45) | 98.7 | 0 | N |
| 37 | + | 18765 | 19331 | 188 | 20.7 | 4.3 | ATG | Phage scaffolding protein [IPR009636; SCAF; Capsid assembly scaffolding protein] | *S. equinus* (5e-130) | 99.5 | 0 | N |
| 38 | + | 19346 | 20233 | 295 | 31.9 | 5.1 | ATG | Phage major capsid protein | *S. equinus* (0.0) | 100 | 0 | Y |
| 39 | + | 20235 | 20495 | 86 | 9.8 | 4.9 | ATG | Hypothetical protein | *S. infantarius* subsp.  *infantarius* (2e-51) | 98.8 | 0 | N |
| 40 | + | 20526 | 20918 | 130 | 14.8 | 4.7 | ATG | Hypothetical protein | *S. equinus* (4e-87) | 99.2 | 0 | N |
| 41 | + | 20908 | 21234 | 108 | 12.1 | 6.9 | ATG | Phage minor capsid protein [IPR019612; Minor_capsid_put; Minor capsid protein, putative] | *S. lutetiensis* (5e-71) | 98.2 | 0 | N |
| 42 | + | 21234 | 21581 | 115 | 12.6 | 10.1 | ATG | Phage minor capsid protein [IPR021080; Minor_capsid_protein; Minor capsid protein] | *S. lutetiensis* (3e-77) | 100 | 0 | N |
| 43 | + | 21581 | 21979 | 132 | 15.0 | 4.6 | ATG | Phage minor capsid protein [IPR024411; Minor_capsid_phage; Minor capsid protein, bacteriophage] | *S. equinus* (2e-90) | 100 | 0 | N |
| 44 | + | 21990 | 22448 | 152 | 16.8 | 4.7 | ATG | Phage tail protein [PF16461; Phage_TTP_12; Lambda phage tail tube protein, TTP] | *S. equinus* (6e-107) | 100 | 0 | N |
| 45 | + | 22462 | 22839 | 125 | 14.5 | 4.5 | ATG | Hypothetical protein | *S. equinus* (5e-86) | 100 | 0 | N |
| 46 | + | 22839 | 23420 | 193 | 22.6 | 5.5 | ATG | Phage protein [IPR009660; Phage_A500_Gp15; Bacteriophage A500, Gp15] | *S. equinus* (5e-86) | 100 | 0 | N |
| 47 | + | 23435 | 27319 | 1294 | 132.4 | 9.3 | ATG | Phage tape measure protein  [PF20155; TMP_3; Tape measure protein] | *S. equinus* (0.0) | 94.3 | 10 | N |
| 48 | + | 27316 | 28803 | 495 | 56.5 | 5.5 | ATG | Phage tail family protein  [PF05709; Sipho_tail; Phage tail protein] | *S. lutetiensis* (0.0) | 98.8 | 0 | N |
| 49 | + | 28804 | 32271 | 1155 | 128.9 | 5.1 | ATG | Collagen-like protein  [IPR007119; Phage_minor_struct_N; Phage minor structural protein, N-terminal domain] | *S. equinus* (0.0) | 80.1 | 0 | N |
| 50 | + | 32288 | 34372 | 694 | 75.2 | 9.3 | ATG | Phage protein [PF05895; DUF859; Siphovirus protein of unknown function] | *S. equinus* (0.0) | 81.8 | 0 | N |
| 51 | + | 34387 | 34770 | 127 | 14.4 | 4.6 | ATG | Phage protein  [PF07104; DUF1366; Protein of unknown function] | *S. equinus* (2e-77) | 91.3 | 0 | N |
| 52 | + | 34748 | 34936 | 62 | 7.2 | 6.8 | ATG | Hypothetical protein | *S. equinus* (2e-35) | 95.2 | 0 | N |
| 53 | + | 34957 | 35289 | 110 | 12.2 | 5.3 | ATG | Hypothetical protein | *S. ruminicola* (8e-71) | 98.2 | 1 | N |
| 54 | + | 35334 | 35657 | 107 | 11.9 | 6.8 | ATG | Phage holin [PF09682; Phage_holin_6_1; Bacteriophage holin of superfamily 6 (Holin_LLH)] | *S. ruminicola* (6e-66) | 96.2 | 1 | N |
| 55 | + | 35657 | 36502 | 281 | 30.8 | 4.8 | ATG | Putative lysin [PF05382; Amidase_5; Bacteriophage peptidoglycan hydrolase] | *S. lutetiensis* (0.0) | 97.2 | 0 | N |
| 56 | + | 36874 | 37035 | 53 | 6.6 | 10.2 | TTG | Hypothetical protein | *S. suis* (3e-08) | 61.8 | 0 | N |
| 57 | + | 37203 | 37589 | 128 | 14.7 | 4.4 | ATG | Hypothetical protein | *S. ruminicola* (2e-86) | 99.2 | 0 | N |
| 58 | + | 37649 | 37999 | 116 | 13.3 | 5.1 | ATG | Hypothetical protein | *S. ruminicola* (4e-74) | 94 | 0 | N |

**Table S4.** PHASTER-based prediction of attachment sites (attL and attR) with >70% nucleotide identity to vB_SbS-proRumen in 27 SBSEC strains identified from 806 publicly available genomes.

| **Bacteria** | **Accession No.** | **PHATEST results** | | **att stie sequence** |
| --- | --- | --- | --- | --- |
|  |  | **attL site region** | **attR site region** |  |
| *S*. *ruminicola* G2 | CP046919.1 | 133065..133081 | 167677..167693 | TGCCCCTTTTTTGCCCC |
| *S*. *equinus* MDC1 | CP059471.1 | - | - | - |
| *S*. *equinus* NM-7 | CP185946.1 | - | - | - |
| *S*. *equinus* Q6 | CP186912.1 | 536852..536864 | 550827..550839 | TTTGTTACGTTTT |
| *S*. *equinus* NCTC8133 | LR594042.1 | 492810..492834 | 505169..505193 | AAATTTGTTACGTTTTTTGTTACGT |
| *S*. *equinus* NCTC8140 | LR134282.1 | 498642..498666 | 511001..511025 | AAATTTGTTACGTTTTTTGTTACGT |
| *S*. *equinus* NM-A1 | CP185763.1 | 1485509..1485522 | 1510833..1510846 | AAGATAAAGAAAAA |
| *S*. *equinus* NM-L6 | CP185947.1 | 1899530..1899541 | 1918030..1918041 | AACATAAAGAAA |
| *S*. *equinus* CNU_77-23 | CP046628.1 | 1588160..1588171 | 1607744..1607755 | TGTAAAAACACC |
| *S*. *lutetiensis* PS.064.S07 | CP145195.1 | 699810..699823 | 739527..739540 | AATATGGTATAATA |
| *S*. *lutetiensis* 033 | CP003025.1 | 1333327..1333342 | 1391751..1391766 | ATTTTCAAATATTTAC |
| *S*. *lutetiensis* FDAARGOS_1018 | CP066277.1 | 194721..194732 | 223792..223803 | AATAATGAAAAT |
| *S*. *lutetiensis* NCTC8738 | LS483348.1 | 685159..685170 | 721414..721425 | CTATCAAGTCAT |
| *S*. *infantarius* FDAARGOS_1019 | CP065994.1 | 399246..399257 | 440796..440807 | AAAATAAGATAA |
| *S*. *infantarius* subsp. *infantarius* CJ18 | CP003295.1 | 782947..782960 | 842986..842999 | GGCAAGAAAAAGTA |
| *S*. *gallolyticus* subsp. *gallolyticus* TX20005 | CP077423.1 | 406313..406327 | 450250..450264 | ATTGTGCTATAATAT |
| *S*. *gallolyticus* FDAARGOS_755 | CP054015.1 | 1208352..1208366 | 1252289..1252303 | ATTGTGCTATAATAT |
| *S*. *gallolyticus* subsp. *gallolyticus* isolate 40 | OZ254909.1 | 2324420..2324434 | 2366551..2366565 | ATTGTGCTATAATAT |
| *S*. *gallolyticus* 202 | CP191581.1 | 354047..354061 | 392663..392677 | ATTGTGCTATAATAT |
| *S*. *gallolyticus* subsp. *gallolyticus* isolate 42 | OZ254911.1 | 407972..407986 | 449533..449547 | ATTGTGCTATAATAT |
| *S*. *gallolyticus* subsp. *gallolyticus* isolate 44 | OZ254912.1 | 122548..122561 | 144478..144491 | AAAAAAAGTAAAAG |
| *S*. *macedonicus* CIP 105683 | CP119172.1 | 662476..662488 | 701083..701095 | TGGTGATTGATTT |
| *S*. *macedonicus* ACA-DC 198 | HE613569.1 | 302491..302510 | 338008..338027 | TGTCAACTGTAGTGGGTGAC |
| *S*. *pasteurianus* 205 | CP191803.1 | 1513389..1513400 | 1543506..1543517 | TTTTTAAACGAA |
| *S*. *pasteurianus* JCM12261 | AP042438.1 | 1051863..1051876 | 1090684..1090697 | ACAAAAAAAGCTCA |
| *S*. *pasteurianus* NCTC13784 | LS483462.1 | 112504..112515 | 154641..154652 | GCGAAAAGAGCT |
| *S*. *pasteurianus* k46-0107-A9 | AP031455.1 | 1147555..1147566 | 1203493..1203504 | GAAATAAAATTA |

**Table S5.** Features of the representative *Streptococcus* phages used in this study.

| **Phage** | **Bacterial host strain** | **Isolation source** | **Genome** | | | |
| --- | --- | --- | --- | --- | --- | --- |
|  |  |  | **Size (bp)** | **Numbers of CDS** | **Annotated ORFs of predicted lysogenic function** | **Accession No.** |
| vB_SbS-proRumen | *S*. *ruminicola* KCCM 90384 | Prophage | 38,092 | 56 | ORF1, integrase;  ORF4, repressor;  ORF19, anti-repressor | PP793892 |
| Javan206 | *S*. *equinus* JB1 | Prophage | 37,284 | 50 | ORF1, integrase;  ORF4, CI-like repressor;  ORF18, antirepressor protein | MK448874 |
| Javan210 | *S*. *equinus* MPR4 | Prophage | 38,639 | 52 | ORF1, integrase  ORF4, CI-like repressor;  ORF19, antirepressor protein | MK448875 |
| Javan284 | *S*. *lutetiensis* 33 | Prophage | 37,997 | 51 | ORF36, antirepressor protein;  ORF48, CI-like repressor;  ORF51, integrase; | MK448898 |
| Javan224 | *S*. *gallolyticus* DD02 | Prophage | 38,781 | 59 | ORF7, antirepressor protein;  ORF22, recombinase | MK448878 |
| SA01 | *S*. *anginosus* SA01 | Human saliva | 36,088 | 53 | ORF3, CI-like repressor | MT900488 |
| Javan83 | *S*. *anginosus* SRR446556 | Prophage | 36,493 | 53 | ORF1, recombinase;  ORF3, CI-like repressor | MK448831 |
| MissD | *S*. *mitis* | - | 38,511 | 62 | ORF15, CI repressor | OL774873 |
| Javan88 | *S*. *canis* ERR438797 | Prophage | 40,047 | 59 | ORF1, integrase;  ORF5, CI-like repressor | MK449009 |
| Javna91 | *S*. *canis* ERR438806 | Prophage | 38,343 | 56 | ORF3, CI-like repressor | MK448834 |
| A25 | *S*. *pyogenes* ATCC 12204 | Sewage | 33,900 | 46 | ORF1, cro family anti-repressor | KT388093 |
| A1 | *S*. *pyogenes* ATCC 12202-B1 | Sewage | 37,239 | 52 | ORF52, recombinase family protein ORF3, CI-like repressor  ORF5, cro family anti-repressor | MW495853 |
| Str01 | *S*. *pyogenes* | - | 37,030 | 54 | ORF25, integrase | KY349816 |
| P9852 | *S*. *thermophilus* ST64985 | Whey | 34,638 | 44 | - | KY705285 |
| 2972 | *S*. *thermophilus* | Yoghurt | 34,704 | 44 | - | AY699705 |
| CHPC1057 | *S*. *thermophilus* | Yoghurt | 34,845 | 45 | - | MH937498 |
| CHPC1008 | *S*. *thermophilus* | Cheese | 34,844 | 46 | - | MH937484 |

**Table S6.** Features of the shared genes between SBSEC temperate phage vB_SbS-proRumen and five representative *Streptococcus* phages (or prophage regions) used in this study.

| ***Streptococcus* phage^*^** | **Shared Gene^**^** | **Protein** | **Accession No.** |
| --- | --- | --- | --- |
| Javan 210, Javan 224,  Javan 206, Javan 284, A1 | TMP_BPSPP | Tail tape measure protein gp18 | Q0PDK7 |
|  | CISA_BACSU | Putative DNA recombinase | P17867 |
|  | YIM3_BPPH1 | Uncharacterized immunity region protein 3 | P10427 |
|  | TNR7_ENTFL | Transposon Tn917 resolvase | P06693 |
|  | XTMA_BACSU | PBSX phage terminase small subunit | P39785 |
|  | TERL_BPSPP | Terminase, large subunit | P54308 |
|  | VSP1_BPLLH | Structural protein | Q04765 |
|  | VSP1_BPMV4 | Structural protein | Q04766 |
| Javan 210, Javan 224,  Javan 206 | ERF_BPP22 | Essential recombination function protein | P04892 |
|  | Y1418_HAEIN | Uncharacterized protein HI_1418 | P44189 |
|  | VF201_IIV6 | Putative Bro-N domain-containing protein 201R | Q91FW9 |
|  | VF201_IIV3 | Putative Bro-N domain-containing protein 019R | Q197E1 |
|  | TERS_BPSPP | Terminase small subunit | P54307 |
|  | TERS_BPSF6 | Terminase small subunit | P68928 |
| Javan 210, Javan 224,  Javan 206, Javan 284 | SSB1_STRA3 | Single-stranded DNA-binding protein 1 | P66850 |
|  | SSB_STRPN | Single-stranded DNA-binding protein | P66854 |
| Javan 210, Javan 224,  Javan 284 | SSB_STRP3 | Single-stranded DNA-binding protein | P0DF76 |
|  | SSB2_STRP8 | Single-stranded DNA-binding protein 2 | Q8NZJ0 |
| Javan 210, Javan 224,  Javan 206 | ALYS_BPDP1 | Lysin | O03979 |
|  | Y1410_HAEIN | Uncharacterized protein HI_1410 | P44184 |
| Javan 210, Javan 206 | LEXA_COLP3 | LexA repressor | Q48AA9 |
|  | YO12_BPHC1 | Uncharacterized 18.2 kDa protein in rep-hol intergenic region | P51714 |
| Javan 206 | CO5A2_HUMAN | Collagen alpha-2(V) chain | P05997 |
|  | CO5A2_MOUSE | Collagen alpha-2(V) chain | Q3U962 |
| Javan 210, Javan 206,  Javan 284 | SCAF_BPLLH | Capsid assembly scaffolding protein | Q04763 |
| Javan 224, Javan 284 | SSB_STRMU | Single-stranded DNA-binding protein | Q8DSD8 |

^*^Five different *Streptococcus* phages that have genes overlapping compared to the phage vB_SbS-proRumen

^**^Genes annotated based on the UniProKB/Swiss-Prot database

**Supplementary figures**


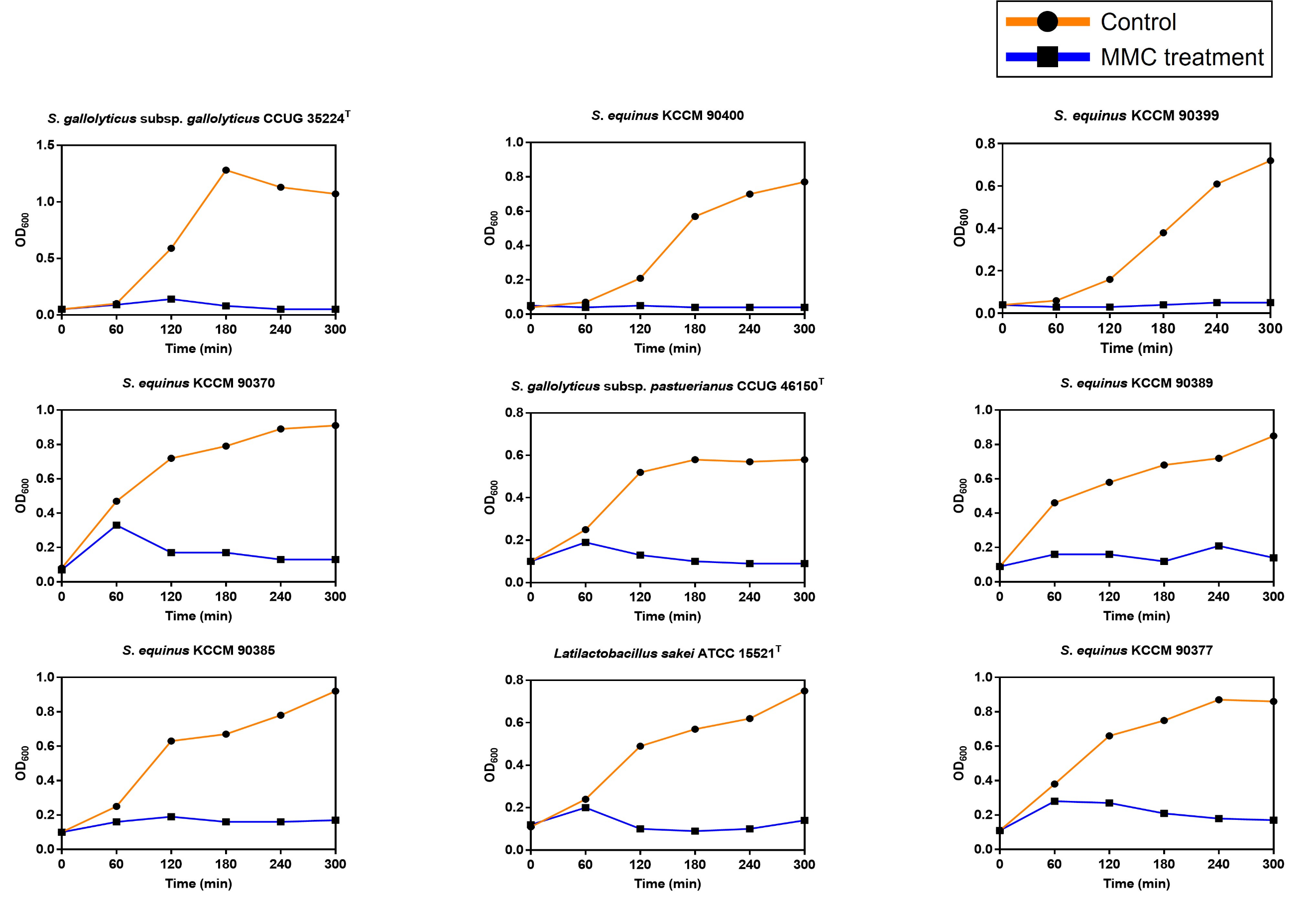


**Fig. S1.** Phage induction profiles of bacterial strains showing complete lysis upon prophage induction. The OD_600_ values of mitomycin C (MMC)-treated and untreated (control) cultures are measured hourly for 5 h. Complete lysis is determined as a reduction of > 80% in OD_600_ values of the MMC-treated group compared to the untreated group at the 5 h time point.


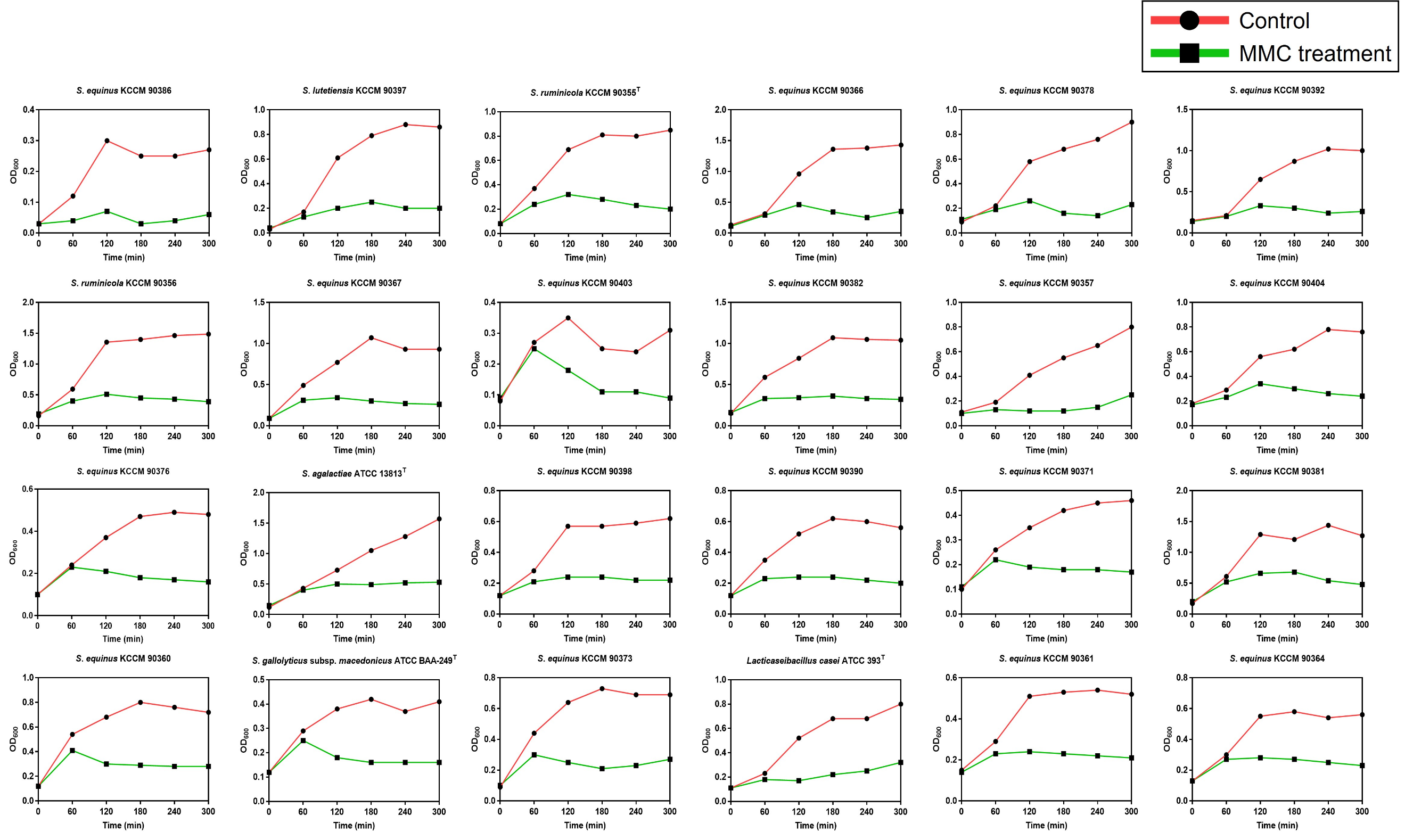


**Fig. S2.** Phage induction profiles of bacterial strains showing partial lysis upon prophage induction. The OD_600_ values of mitomycin C (MMC)-treated and untreated (control) cultures are measured hourly for 5 h. Partial lysis is determined as a reduction of 60–80% in OD_600_ values of the MMC-treated group compared to the untreated group at the 5 h time point.


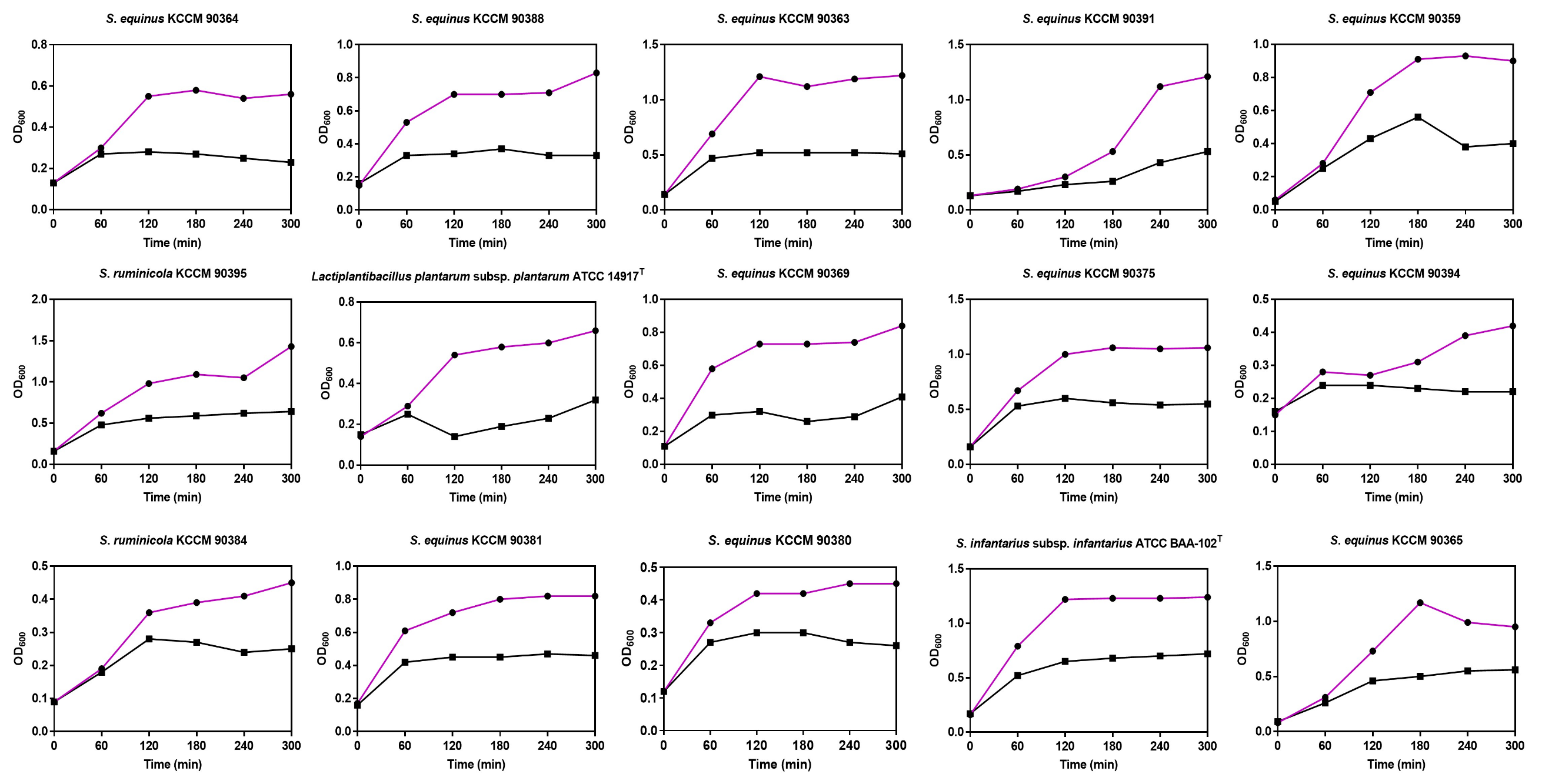


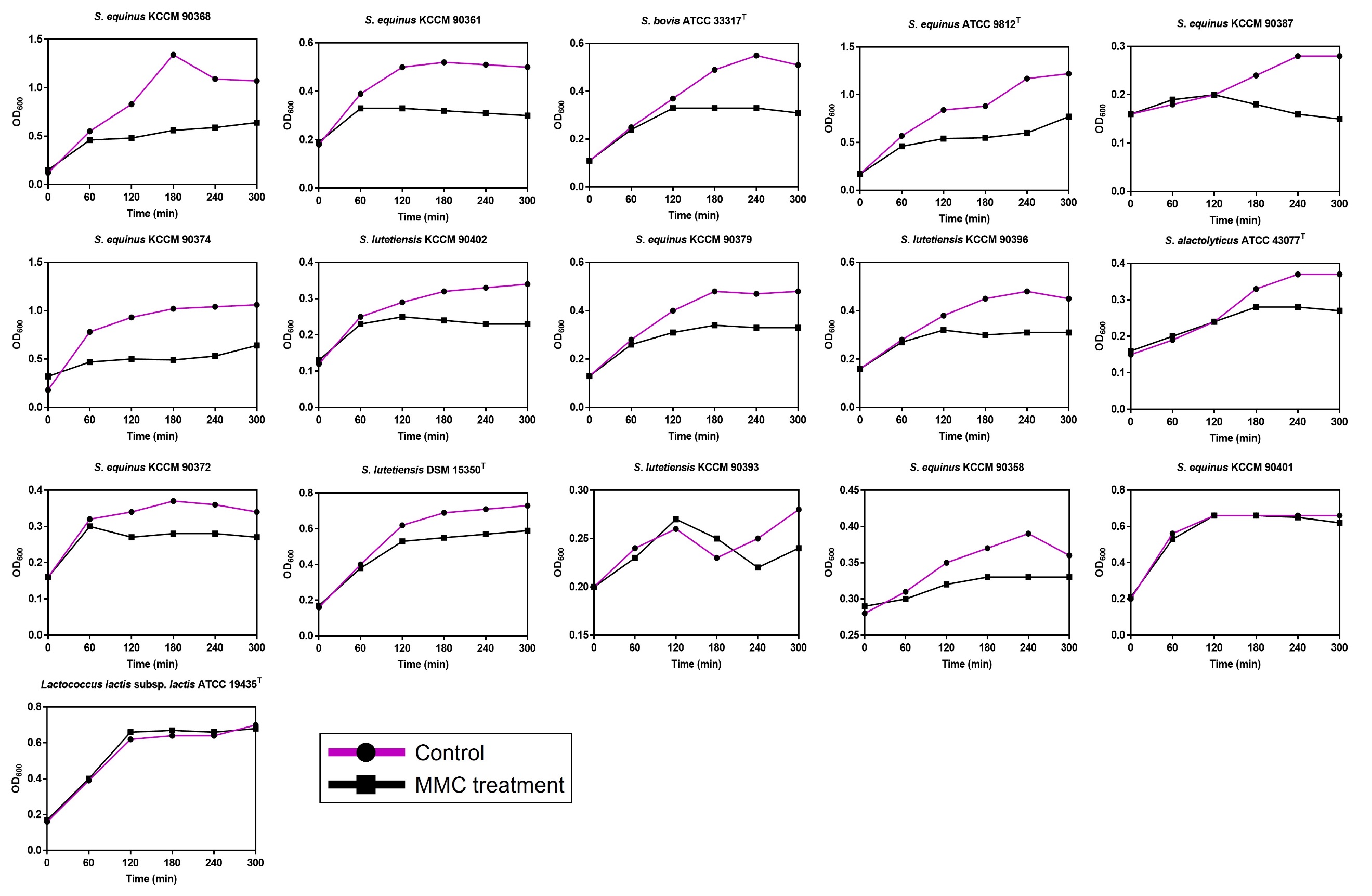


**Fig. S3.** Phage induction profiles of bacterial strains showing no lysis upon prophage induction. The OD_600_ values of mitomycin C (MMC)-treated and untreated (control) cultures are measured hourly for 5 h. No lysis is determined as a reduction of < 60% in OD_600_ values of the MMC-treated group compared to the untreated group at the 5 h time point.


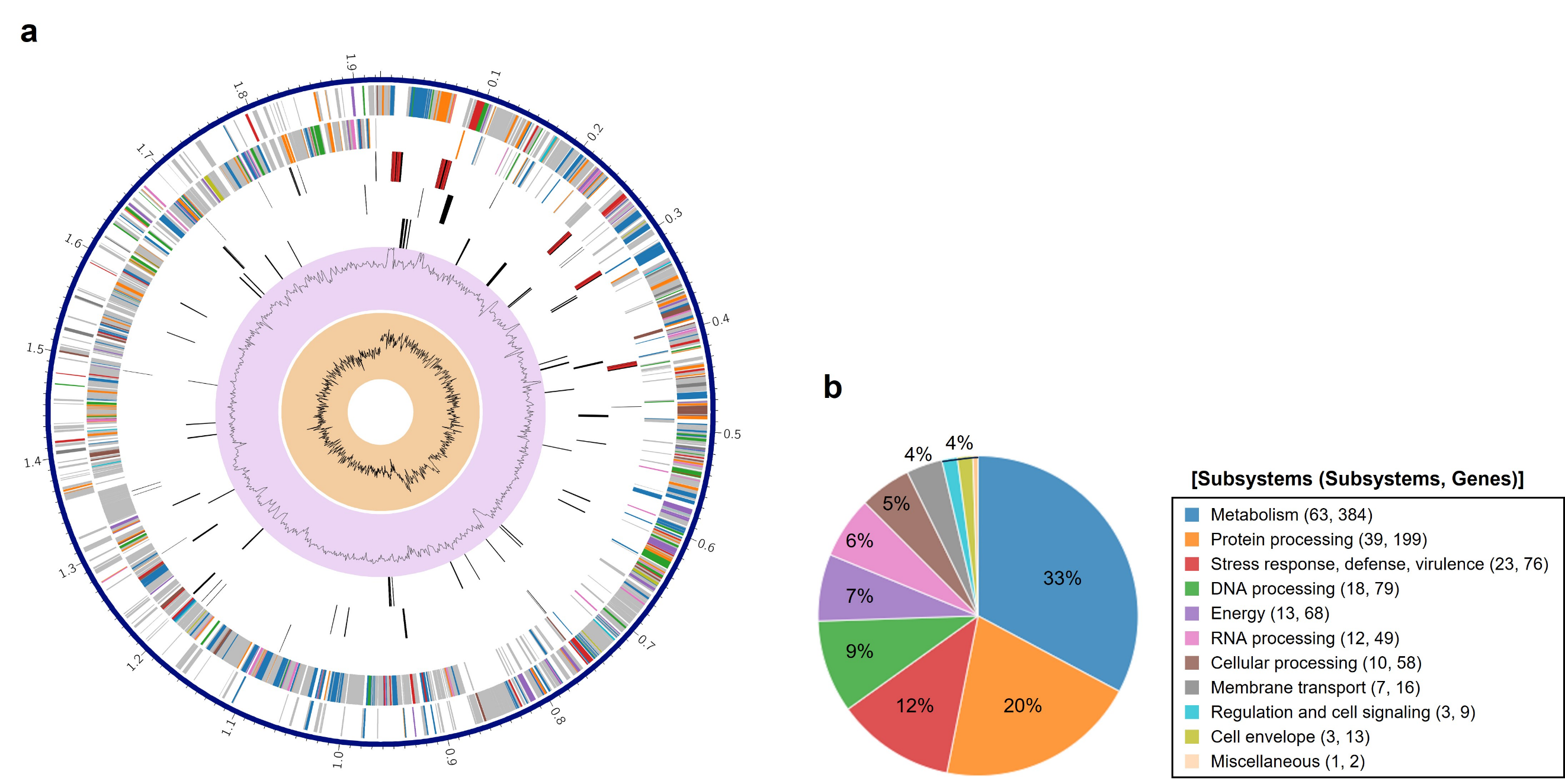


**Fig. S4.** Circular genome map and subsystem analysis of lysogenic *S. ruminicola* KCCM 90354. Genomic map **(a)** of strain KCCM 90354 generated using PATRIC. The outer number markers indicate nucleotide positions in base pairs (bp). From the outermost ring to the center: ring 1 (forward strand), coding sequences (CDSs) on the forward strand colored by subsystem category; ring 2 (reverse strand), CDSs on the reverse strand colored by subsystem category; ring 3, non-coding RNAs (tRNAs and rRNAs); ring 4, GC content; ring 5, GC skew. **(b)** Subsystem analysis of strain KCCM 90354 generated using PATRIC, representing the distribution of annotated genes across specific biological processes and structural complexes. The values in parentheses indicate the number of subsystems and the number of genes assigned to each category, respectively.


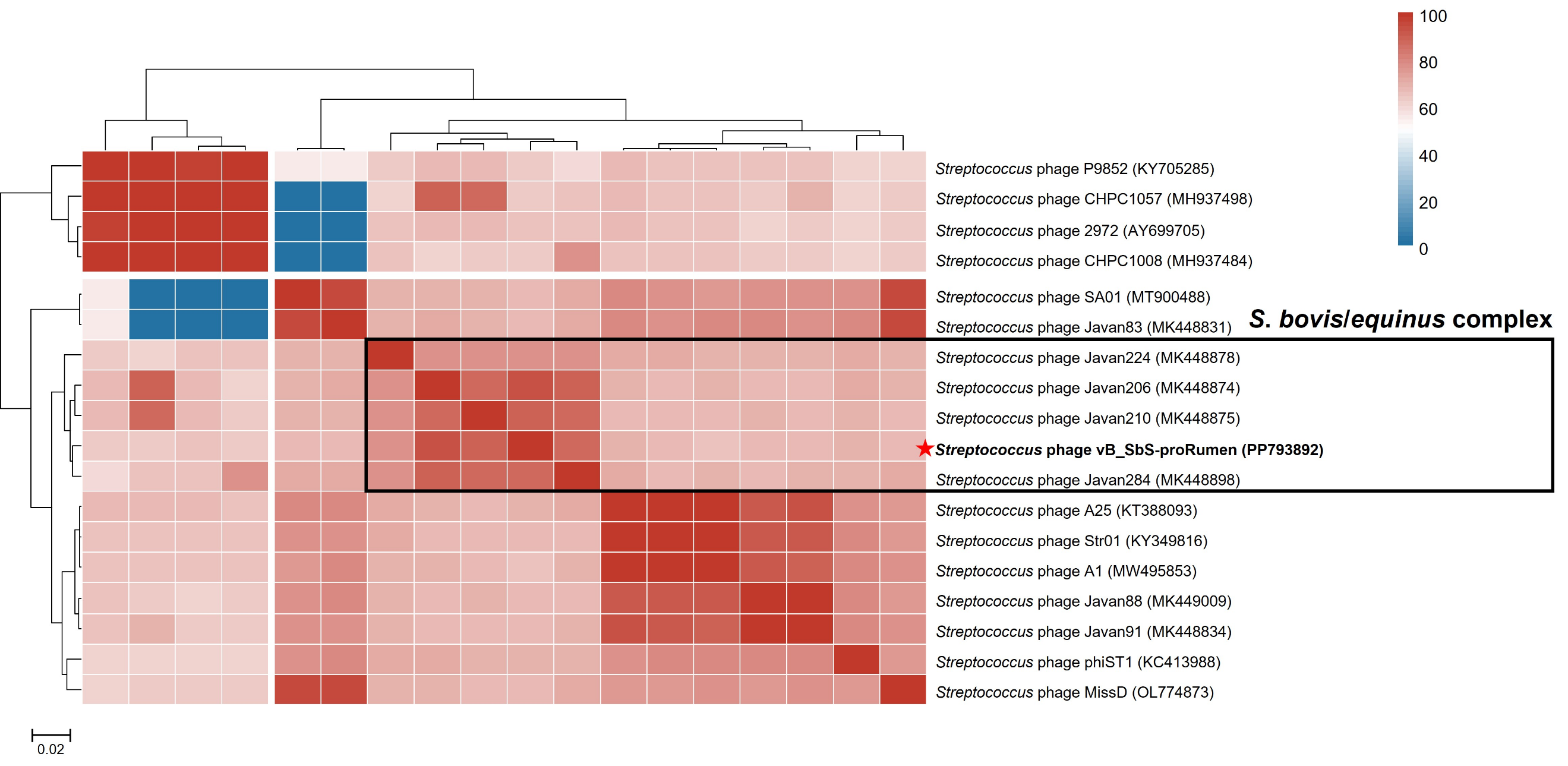


**Fig. S5.** Heatmap of SBSEC temperate phage vB_SbS-proRumen based on orthoANI values using the ‘pheatmap’ R package. The map is constructed to illustrate the genomic relationship between vB_SbS-proRumen and 17 closely related *Streptococcus* phages (or prophages), revealing distinct clusters formed by intergenomic similarities. The phage used in this study is highlighted in bold.


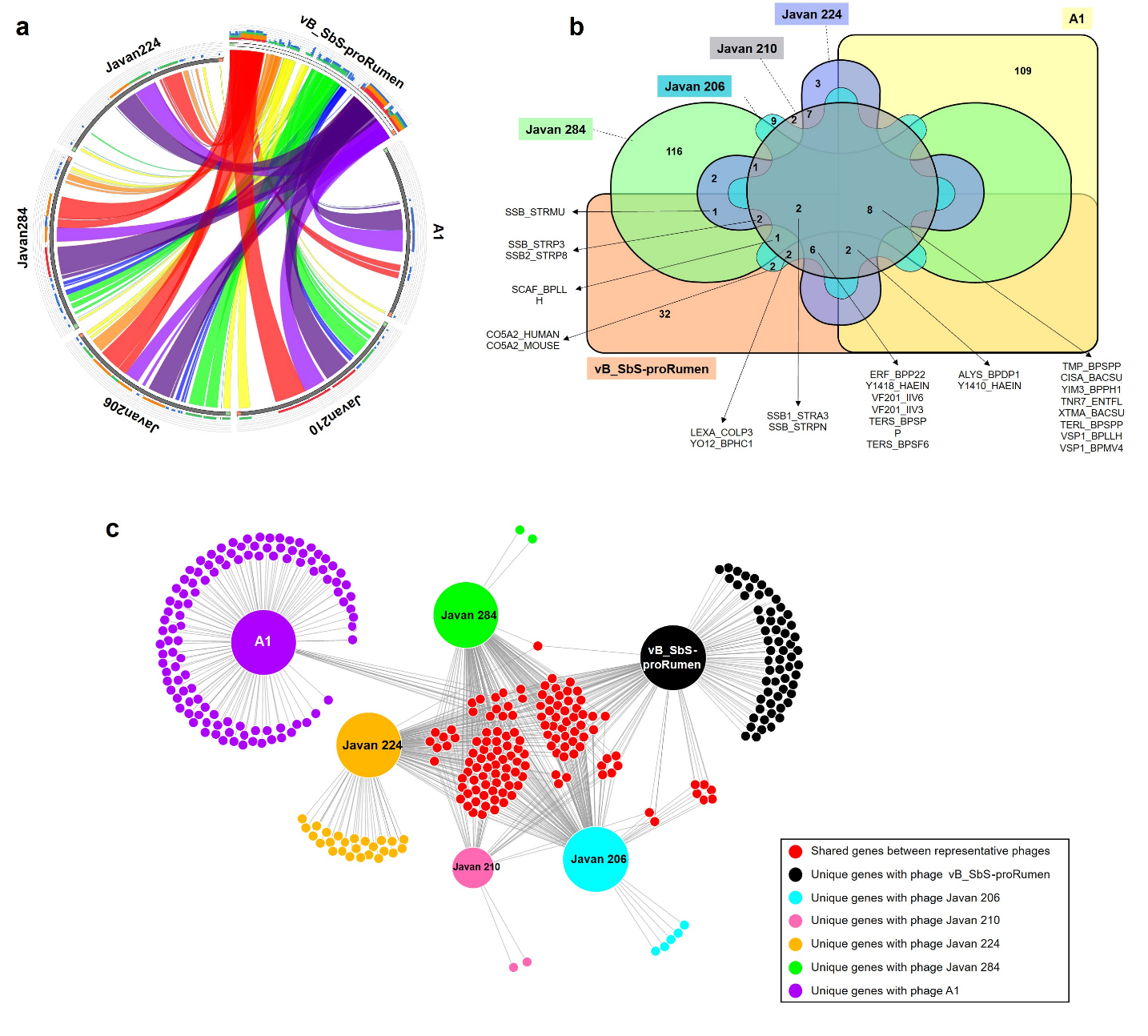


**Fig. S6.** Genome-based comparison of SBSEC temperate phage vB_SbS-proRumen with predicted genomic regions in its homologous prophages; *Streptococcus* prophages Javan 210 (MK448875), Javan 206, (MK448874), Javan 284 (MK448898), Javan 224 (MK448878), and A1 (MW495853). **(a)** Synteny map linking the genomes of vB_SbS-proRumen and representative prophages illustrated by colored ribbons based on the best BLAST alignment score using Circoletto. **(b)** Venn diagram showing the number of overlaps between protein-coding genes asin vB_SbS-proRumen and representative prophages constructed using InteractiVenn. The unique and shared genes are annotated using the UniProtKB/Swiss-Prot database; detailed descriptive protein names and corresponding accession codes are provided in Table S6. **(c)** DiVenn diagram depicting up- (red) and down-regulated (blue) unique and shared proteins in vB_SbS-proRumen and representative prophages generated by DiVenn. Red and blue nodes indicate genes that are present or absent, respectively, in vB_SbS-proRumen relative to the compared prophage genomes.
